# Sesquiterpene emissions from *Alternaria alternata* and *Fusarium oxysporum*: Effects of age, nutrient availability, and co-cultivation

**DOI:** 10.1038/srep22152

**Published:** 2016-02-26

**Authors:** Fabian Weikl, Andrea Ghirardo, Jörg-Peter Schnitzler, Karin Pritsch

**Affiliations:** 1 Helmholtz Zentrum München - German Research Center for Environmental Health, Institute of Biochemical Plant Pathology (BIOP), Neuherberg, Germany; 2 Helmholtz Zentrum München - German Research Center for Environmental Health, Research Unit Environmental Simulation (EUS), Institute of Biochemical Plant Pathology, Neuherberg, Germany

## Abstract

*Alternaria alternata* is one of the most studied fungi to date because of its impact on human life – from plant pathogenicity to allergenicity. However, its sesquiterpene emissions have not been systematically explored. *Alternaria* regularly co-occurs with *Fusarium* fungi, which are common plant pathogens, on withering plants. We analyzed the diversity and determined the absolute quantities of volatile organic compounds (VOCs) in the headspace above mycelial cultures of *A. alternata* and *Fusarium oxysporum* under different conditions (nutrient rich and poor, single cultures and co-cultivation) and at different mycelial ages. Using stir bar sorptive extraction and gas chromatography–mass spectrometry, we observed *A. alternata* to strongly emit sesquiterpenes, particularly during the early growth stages, while emissions from *F. oxysporum* consistently remained comparatively low. The emission profile characterizing *A. alternata* comprised over 20 sesquiterpenes with few effects from nutrient quality and age on the overall emission profile. Co-cultivation with *F. oxysporum* resulted in reduced amounts of VOCs emitted from *A. alternata* although its profile remained similar. Both fungi showed distinct emission profiles, rendering them suitable biomarkers for growth-detection of their phylotype in ambient air. The study highlights the importance of thorough and quantitative evaluations of fungal emissions of volatile infochemicals such as sesquiterpenes.

Sesquiterpenes are a class of highly reactive volatile terpenoids (C_15_H_24_). They function as infochemicals^1^,^2^ and play crucial roles in plant-to-plant, plant-to-microbe/animal and microbe-to-microbe interactions^3^. Industrially, sesquiterpenes can act as precursors of advanced biofuels with properties similar to petroleum-based fuels^4^,^5^,^6^,^7^. Sesquiterpenes can affect atmospheric chemistry and impact climate in a similar manner to other volatile terpenoids^8^,^9^, although – due to analytical difficulties – a large uncertainty exists about sesquiterpene emissions^10^. Microorganisms and especially fungi have recently been recognized as potentially important sources of volatile organic compounds (VOCs)^3^. Thus, studying microbial volatile terpenoids is both industrially valuable and essential to understand biotic and biosphere-atmosphere interactions.

The genus *Alternaria* comprises saprotrophic and plant-pathogenic fungi and includes some ubiquitous species, such as *A. alternata*^11^,^12^. Recently, the genomes of 25 *Alternaria* spp. were sequenced, paving the way for the molecular exploration of their different lifestyles and their underlying metabolomics networks^13^. Studies on metabolites of the genus *Alternaria* mainly focused on agricultural spoilage via mycotoxins^14^ or toxin-mediated plant pathogenicity^15^, while fewer studies targeted VOCs. A recent compilation of original work on microbial VOCs^16^ mostly includes substances for *Alternaria* that are commonly found throughout the fungal kingdom, such as 1-octen-3-ol or 3-octanone. Accounts of sesquiterpene biosynthesis in *Alternaria* are scarce, and of 268 metabolites reported for the genus^17^, just two were sesquiterpene-derived compounds, commonly called ‘oxygenated sesquiterpenes’ (oSQT).

In addition to its role as a plant pathogen^11^ and producer of mycotoxins^14^, *A. alternata* is a major fungal allergen source^18^, which has led to it becoming one of the most thoroughly studied fungi. Still, the microbial VOC database^19^ lists only one compound (6-methoxyheptanol) for *A. alternata*, which is the only representative of the genus in the database (accessed 20/09/15). However, sesquiterpenes have been reported for *A. alternata*^20^ and two compounds ((+)-β-cedrene and (−)-thujopsene) have been identified.

Knowledge about sesquiterpene emissions is greater for some species of *Fusarium* compared to *Alternaria*. Eighteen different sesquiterpenes were identified in two strains of *F. sambucinum* and one strain of *F. sporotrichioides*, as well as no or six sesquiterpenes in two different strains of *F. graminearum*^21^, respectively.

As previously mentioned, sesquiterpenes can act as biological infochemicals, and several examples of volatile-mediated interactions have been described for *Fusarium*. Volatiles (including sesquiterpenes) from a non-pathogenic *Fusarium oxysporum* strain suppressed the growth and gene expression of plant pathogenic strains of this species^22^. Other strains of *F. oxysporum* with different VOC profiles have been shown to inhibit the growth of nematodes^23^ or fungal pathogens^24^.

Most of these findings have been recently made because the analytical techniques for the detection of a broad spectrum of VOCs have constantly improved over the last two decades^25^. Headspace sampling and stir bar sorptive extraction (SBSE) coupled to gas chromatography–mass spectrometry (GC-MS) have recently been applied for *in vitro* ecotyping of fungi based on their volatile profiles^26^. The same method was used to reveal reprogramming of root architecture through sesquiterpene signaling, thus highlighting the role of sesquiterpenes in plant-microbe interactions^1^. However, absolute quantification of sesquiterpene emissions that would allow comparisons between studies is rare in fungal VOCs research.

In this study, we aimed to comprehensively identify and quantify sesquiterpene production from *A. alternata* and *F. oxysporum* as a function of the growth stage, nutrient conditions, and fungus-fungus interactions. With investigating the variability of fungal sesquiterpene emissions, we provide fundamental data for exploring the different ecological functions of fungal VOCs i.e., sesquiterpenes related to the different lifestyles and for applied approaches such as the use of sesquiterpene biomarkers for fungi.

## Results

### Growth characteristics

**Figure 1:**
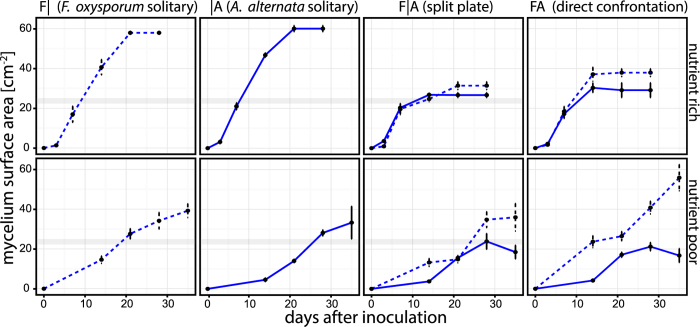
Mycelial expansion during VOC sampling. Upper row: nutrient rich (malt extract gelrite media), lower row: nutrient poor (synthetic nutrient poor gelrite media); solid line: *Alternaria alternata*, dashed line: *Fusarium oxysporum*; points and error bars depict means ± s.d. (n = 5) of mycelium areas at VOC sampling time-points. Grey strips: approximate size of mycelia when hyphae started to grow across the glass-barriers separating the gelrite hemispheres of the plates.

Under nutrient rich conditions, the mycelia of both fungi grew similarly with growth ceasing between day 7 and 14 (co-cultivation) or day 14 and 21 (solitary cultivation). Neither fungus was able to overgrow the other, but *F. oxysporum* covered most of the glass-barriers on split-plates as it grew onto them more quickly. Cultures were aged after day 28, as indicated by partly collapsed and hyaline hyphae. (Figure 1; morphologies of nutrient-rich split-plates are shown in Supplementary Fig. S1).

Under nutrient poor conditions*, F. oxysporum* grew faster than *A. alternata*; however, the growth of *F. oxysporum* under co-cultivation slowed between day 14 and day 21. Between days 21 and 28, *F. oxysporum* started to overgrow peripheral parts of *A. alternata* mycelia. At day 35, both fungi were still growing when cultivated alone. (Figure 1; morphologies of nutrient poor conditions are shown in Supplementary Fig. S2).

No drying of the media in the sealed petri-dishes was observed. Small condensation droplets were commonly formed on the lids of the plates after each resealing, indicating high relative humidity throughout the sampling period.

### Overview of VOC emission diversity and strength

The chemical compounds in VOC emissions were quantitatively and qualitatively diverse, and the quantity was higher for *A. alternata* compared to *F. oxysporum*. Altogether, we detected 26 different volatile compounds of unambiguous fungal origin, 24 sesquiterpenes and two alcohols (Table 1, Supplementary Tables S1–S4). From *A. alternata*, 25 compounds were emitted – in total up to 183 ± 82 pmol cm^−2^ h^−1^ (day 3, nutrient rich), while *F. oxysporum* emitted 10 compounds – in total up to 6.7 ± 4.7 pmol cm^−2^ h^−1^ (day 3, nutrient rich), both referring to the mycelium area. All VOCs from *F. oxysporum* were also detected in *A. alternata*, except for δ-elemene (Table 2).

**Figure 2:**
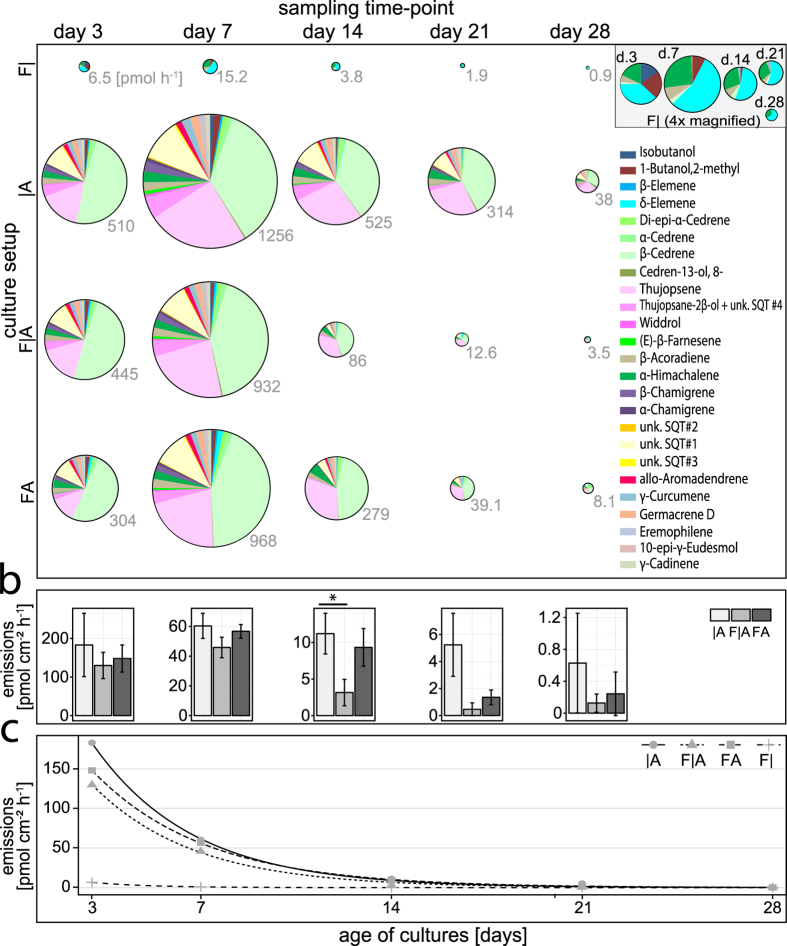
Time-course of VOC emission rates from fungi growing under nutrient rich conditions. (**a**) Total VOC emission rates under different cultivation conditions; **F|**: *Fusarium oxysporum* alone, |**A**: *Alternaria alternata* alone, **F|A**: split-plate with both fungi, **FA**: direct confrontation of both fungi; pie chart areas are proportional to the emission rate per culture plate, grey numbers: means (n = 5) of total VOC emission rate (pmol plate^−1^ h^−1^); the exact values underlying each pie chart are available in Supplementary Tables S1–S2. (**b**) Total VOC emission rate normalized to the projected mycelium area (pmol cm^−2^ h^−1^) of *A. alternata* on each plate (**F|A** and **FA** setups: ignoring the minute share of *F. oxysporum* on total emissions); means ± s.e. (n = 5); *P = 0.04, t-test. (**c)** Total VOC emissions from (**b**) as function of the culture age, including emissions from **|F** which were normalized to the projected mycelium area of *F. oxysporum*.

**Figure 3:**
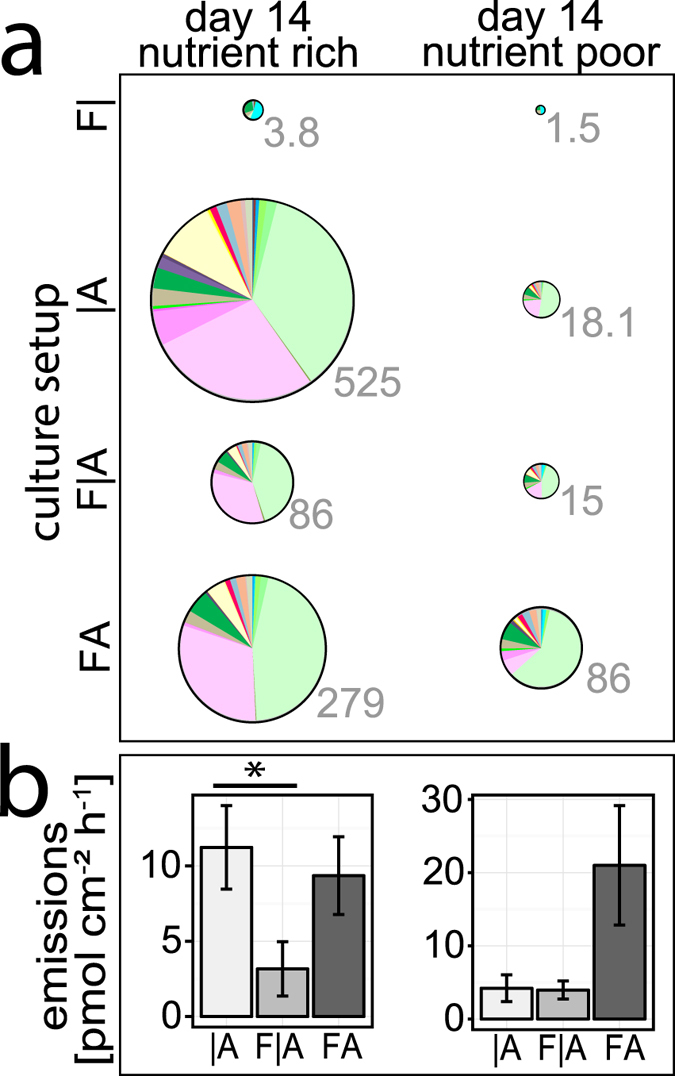
Emission rate after 14 days of cultivation on nutrient rich and poor media. (**a**) Total emission rate of VOC under different cultivation conditions; **F|**: *Fusarium oxysporum* alone, |**A**: *Alternaria alternata* alone, **F|A**: split-plate with both fungi, **FA**: direct confrontation of both fungi; pie chart areas are proportional to the emitted volatiles, grey numbers: means (n = 5) of total VOC emission rates (pmol plate^−1^ h^−1^); the exact values underlying each pie chart are available in Supplementary Tables S1–S2. Colors are coded as in Fig. 2a. (**b**) Total emission rate normalized to the projected surface area (pmol cm^−2^ h^−1^) of *A. alternata* on each plate (**F|A** and **FA** culturing: ignoring the minute share of *F. oxysporum* on the total emissions); means ± s.e. (n = 5).

All 26 substances were detected when both fungi grew in direct contact (direct confrontation plates) under nutrient rich conditions. Some compounds were not detected when the fungi grew alone, or were physically separated (split-plates) (Supplementary Tables S1–S2), or were grown under nutrient poor conditions (Table 2). Solitary cultures of *A. alternata*, split-plate cultures, and direct confrontation cultures of the same age differed only in the compounds that were present in the lowest amounts. The emission profiles were highly similar between *A. alternata* grown alone and co-cultivated with *F. oxysporum*, except for the appearance of δ-elemene in the co-cultivation (Figs 2a and 3a).

Under nutrient rich conditions, the emission rate per plate was strongest at day 7, while the emission rate per mycelium area was strongest at day 3 (Fig. 2). Under nutrient poor conditions, the emission rate was highest at day 14 (Supplementary Tables S1 and S3), the first time-point measured.

Under nutrient rich conditions, the average emission rate per mycelium area of *A. alternata* grown alone was more than an order of magnitude higher than those of *F. oxysporum* (e.g., 27 times more at day 3, 60 times more at day 7, and 122 times more at day 14; Supplementary Table S3). Similar differences were obtained under nutrient poor conditions (e.g., 51 times more at day 14 and 85 times more at day 21; Supplementary Table S3). The total emission intensity of *A. alternata* decreased exponentially with culture age (Fig. 2c).

The emission rate per mycelium area from nutrient rich split-plates at day 14 of co-cultivation were significantly lower than those of the same age of *A. alternata* grown alone (t-test *P* = 0.04) (Fig. 2). Over all cultivation conditions and all time-points, the emission rate (pmol cm^−2^ h^−1^) decreased in the order: *A. alternata* alone > direct confrontation > split-plate > solitary *F. oxysporum*.

The emission rate (day 14) in solitary cultures of *A. alternata* and co-cultivations with *F. oxysporum* were much higher under nutrient rich than nutrient poor conditions, when calculated per plate (pmol h^−1^) (Fig. 3a). However, those differences became insignificant (t-tests *P* > 0.05) when calculated based on the mycelium area of *A. alternata* (Fig. 3b).

In contrast, the emission rate of solitary *F. oxysporum* under nutrient rich and poor conditions were roughly similar when calculated per plate (3.8 vs. 1.5 pmol h^−1^; Fig. 3a) as well as when based on the mycelium area of *F. oxysporum* (0.092 vs. 0.082 pmol cm^−2^ h^−1^; Supplementary Table S3).

### Emission of specific compounds

Under all nutrient conditions and at all time-points, the sesquiterpenes thujopsene and β-cedrene were the most abundant VOCs of *A. alternata*. Together, they constituted more than two-thirds of the total emissions (Fig. 2). The next highest emissions generally were an unknown SQT (sesquiterpene) #1, a mixture of thujopsane-2β-ol plus unknown SQT #4, α-himachalene, and β-acoradiene (Table 1, Supplementary Fig. S3). The general emission patterns of *A. alternata* did not differ notably with culture age, but the relative contributions of thujopsene and β-cedrene to the total emissions changed considerably between sampling time-points (Supplementary Table S1).

The emission pattern of the compounds detected for *F. oxysporum* was less constant (detection was approximately at the limit of quantification for some substances) and the overall emission rate was more than 100-fold lower at most time-points than for *A. alternata* (Fig. 2, Supplementary Fig. S4). For example, at day 3 under nutrient rich conditions, seven compounds were detected for *F. oxysporum* (highest: δ-elemene, 2-methyl-1-butanol, and α-himachalene), while three weeks later (day 28), only two compounds (δ-elemene and α-himachalene) remained detectable (Supplementary Table S1). Three substances (γ-curcurmene, germacrene D, and an unknown SQT #2) were only detected in trace amounts (<0.003 pmol cm^−2^ h^−1^) on one or two sampling dates.

Common to all cultures was a much more rapid decline in the emission rate of alcohols compared to sesquiterpenes. By day 14, only traces of alcohols were detected (e.g., 0.04 ± 0.008 pmol cm^−2^ h^−1^ 2-methyl-1-butanol emitted from solitary cultures of *A. alternata*).

Amounts of compounds under nutrient poor cultivation resembled those of the nutrient rich cultivations for *A. alternata* (except for the absent compounds, Table 2). For *F. oxysporum*, emissions were very low (day 14: 0.082 ± 0.051 pmol cm^−2^ h^−1^, day 21: 0.007 ± 0.005 pmol cm^−2^ h^−1^, day 35: 0.001 ± 0.0001 pmol cm^−2^ h^−1^), therefore no temporal trends for the amounts of individual compounds from *F. oxysporum* were inferred for nutrient poor conditions.

**Figure 4:**
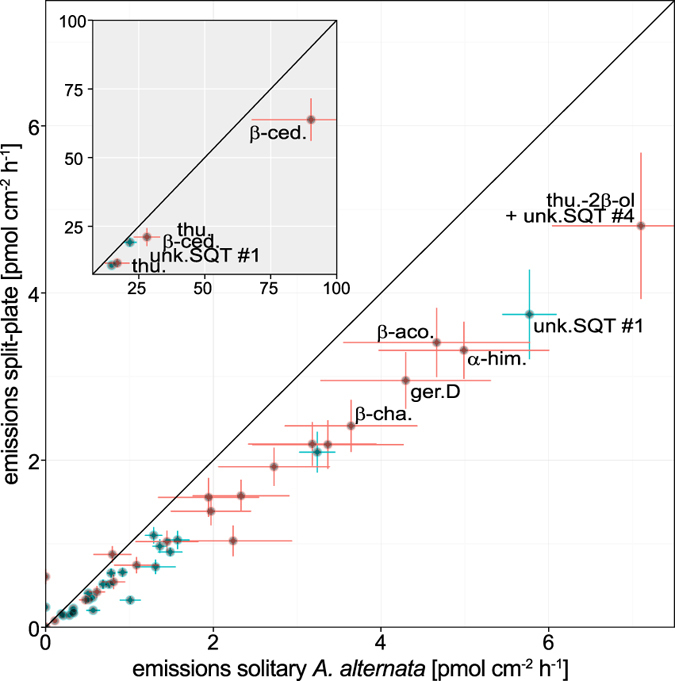
Emission rate per mycelium surface area of *Alternaria alternata* compared between solitary cultures of *A. alternata* and split-plates with the headspace shared by *A. alternata* and *Fusarium oxysporum*. Points with standard errors (n = 5, each): emissions of single compounds, red: day 3 VOC sampling, blue: day 7 VOC sampling; the diagonal line indicates equal results for both setups. The insert shows the results for the strongest emissions on a larger scale. Full names of abbreviated compounds are given in Table 1.

Almost all individual compounds from *A. alternata* were found in lower amounts on split-plates compared to solitary cultivation (Fig. 4). When grown together with *F. oxysporum*, some sesquiterpenes of *A. alternata* were significantly less abundant, e.g., β-cedrene (days 14 and 21; t-tests *P* = 0.001) and thujopsene (days 7 and 14; t-tests *P* = 0.005).

### Multivariate analyses and correlation matrices

**Figure 5:**
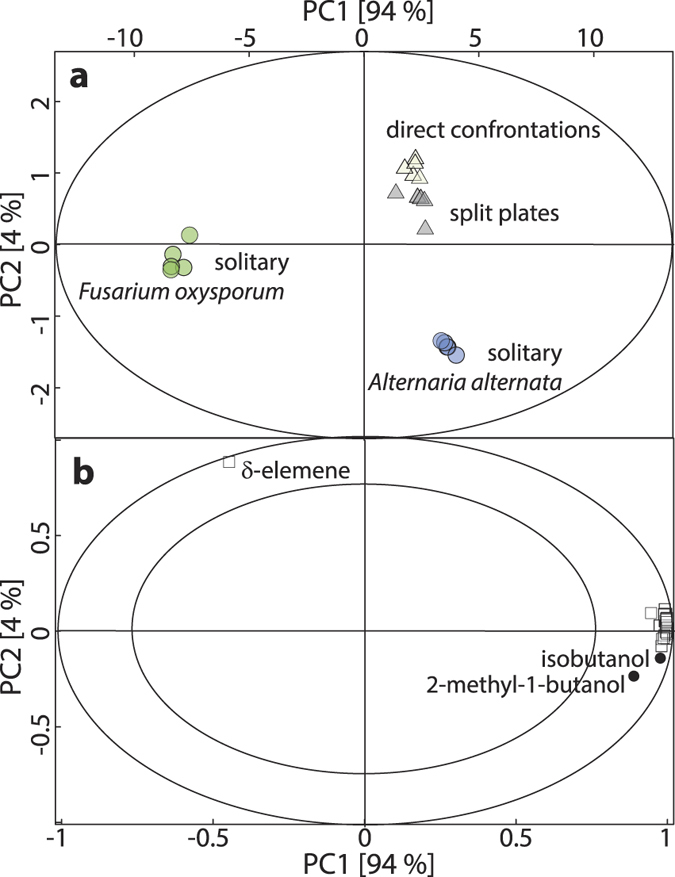
Principal component analysis (PCA) of VOC emission rates of the five biological replicates of all different fungal setups (7-day-old cultures, nutrient-rich media). PC1, PC2: principal first and second components with total explained variance given as percentage. (**a**) Score plot: solitary fungal culture of *Fusarium oxysporum* (green) and *Alternaria alternata* (blue) are depicted with circles; direct confrontations are indicated by beige triangles and split plates by grey triangles; the ellipse indicates the tolerance based on Hotelling’s *T*^*2*^ with a significance level of α = 0.05. (**b**) Correlation scaled loading plot: white squares: sesquiterpenes, black dots: alcohols; the outer and inner ellipses indicate 100% and 75% explained variance, respectively.

VOC profiles strongly differed in multivariate analyses (PCA and OPLS) – in quality and quantity – between *A. alternata* and *F. oxysporum* cultures growing alone, as shown by the large distance and separation between *A. alternata* and *F. oxysporum* samples in the first significant principal component in a PCA, which explained 94% of the total VOC variance (Fig. 5a).

Furthermore, VOC emissions from co-cultivated fungi could be separated from those emitted from cultures containing only one fungus, as demonstrated by the separation of the second significant principal component. PCA indicated differences in emissions between fungi growing in direct contact or on split plates, but these were rather marginal.

Overall, high emission rates of sesquiterpenes were negatively correlated in solitary cultures of *F. oxysporum* (PCA: Fig. 5b, OPLS: Supplementary Fig. S5). Only the sesquiterpene δ-elemene, unique to *F. oxysporum*, was highly and negatively correlated to solitary cultures of *A. alternata* with a scaled and centered regression coefficient of −0.82 in the OPLS model (Supplementary Fig. S5).

The OPLS model described changes of VOC emissions from the different fungal cultures very well; the cumulative *R*^*2*^(X) and *Q*^*2*^(cum) were 98.6% and 70.1% respectively, using 3 predictive components. The analysis of variance testing the cross-validated predictive residuals (CV-ANOVA) indicated that the OPLS model discriminated the cultures of a single fungus in a highly reliable manner (*P*-values: *A. alternata* <2 × 10^−7^, *F. oxysporum* <4 × 10^−14^, direct confrontation = 0.0499). Regression lines for observed versus predicted Y-values were *R*^*2*^ = 0.955 (*A. alternata*) and *R*^*2*^ = 0.996 (*F. oxysporum*). Classification exercises indicated that an OPLS model based on VOC emission profiles collected at day 7 can correctly predict the presence of a solitary culture of *A. alternata* and *F. oxysporum* at day 3, 7, 14 (threshold set to 0.5 predicted Y values) (Supplementary Fig. S5).

Spearman correlation matrices that included all time-points of solitary cultivations showed positive correlations (Spearman *P* < 0.05, Supplementary Fig. S6) for emission rates of most compounds for each fungus. Nearly half of the sesquiterpenes produced by *A. alternata* were almost totally correlated (Spearman correlation coefficient *ρ* > 0.9), as were δ-elemene and α-himachalene for *F. oxysporum* (*ρ* = 0.97).

## Discussion

Our sampling and detection system allowed the detailed assessment of sesquiterpene formation in the two fungi. The quantitative differences in VOC emissions between them were very distinct; less abundant compounds from *A. alternata* were still emitted at the same magnitude as the most abundant VOC released from *F. oxysporum*.

As observed in this study, *A. alternata* had the highest sesquiterpene emissions relative to other fungi in an earlier study^20^. However, direct comparison with our data is impeded by the lack of absolute quantification in most of the earlier studies. A recent study^26^ applied the same detection system with sealed plates and a synthetic medium comparable to the nutrient poor medium that we used in this study. That study analyzed emission profiles and source strengths from eight fungi of different ecotypes (ectomycorrhizae, pathogens, and saprophytes) and observed the highest sesquiterpene emissions for *Trichoderma viride* (~0.7 pmol cm^−2^ h^−1^ at an age of 3 days). At the first sampling date in our study (day 14), the emission rate for *A. alternata* was still approximately six-fold higher than for *T. viride* in the earlier study, while those for *F. oxysporum* were three times lower. However, the emission rate in nutrient rich medium in this study at day 3 was more than 250 times higher for *A. alternata* and ten times higher for *F. oxysporum*, compared to the earlier data on *Trichoderma viride*.

In plants – a global major source of terpenes^10^ – lower sesquiterpene emission rates were often reported^27^ than the emission rate from *A. alternata* found here. *A. alternata* is geographically widespread and abundant in nature^12^. Therefore, *A. alternata* might potentially contribute significantly to the emission of sesquiterpenes into the atmosphere, as generally hypothesized for fungi^3^.

This study expanded the sesquiterpenes of *A. alternata* described from two major compounds^20^ to a complex variety of substances. We used a passive sampling system specifically suitable to trap non-polar compounds such as sesquiterpenes but with a low affinity for relatively polar compounds such as alcohols, ketones, and aldehydes. Therefore, their contribution to the total VOC emitted from *A. alternata* in the present analysis cannot be completely assessed. Physicochemical differences in the VOC collection system and trapping material can explain why previous studies found different chemical compounds for *Alternaria*^16^.

We detected oSQT as minor constituents in the *A. alternata* emission profile. Few oSQT have been described for fungi so far^28^. To the best of our knowledge, this is the first report on the production of widdrol, thujopsane-2β-ol and cedren-13-ol, 8 in fungi, while isomers of 10-epi-γ-eudesmol have been described for freshwater fungi (*Beltrania rhombica*)^29^ and mushrooms (*Inonotus obliquus*)^30^. Our most predominant VOC, β-cedrene, has only been reported in fungal odor profiles from *A. alternata*^20^ and *Penicillium decumbens*^31^. The structurally related di-epi-α-cedrene and α-cedrene have been found in some other ascomycetes^28^. The second most abundant substance in our study, thujopsene, has been reported in various fungi including *Penicillium decumbens*^31^. This fungus emitted thujopsene in addition to β-cedrene and other compounds, which included substances that were also detected in this study for *A. alternata*, e.g., (*E*)-β-farnesene, β-acoradiene, β-chamigrene, and α-chamigrene. Thujopsene was found to have an auto-regulatory function for the growth of *P. decumbens* and inhibitory effects on several ascomycetes^31^.

Compared to earlier studies on fungal VOCs, the number of sesquiterpenes from *A. alternata* was relatively high. This confirmed results from recent measurements showing that many fungi emit a suite of many different sesquiterpenes^21^,^31^,^32^,^33^. Our attempt to relate sesquiterpene synthases (SQTS) to this multitude of sesquiterpenes by using emission correlation matrices, as has been shown recently^34^, gave ambiguous results and suggested that either the same enzyme was responsible for the formation of all sesquiterpenes or that different SQTS were biochemically active in concert. The formation of multiple products by single sesquiterpene synthases has been documented in fungi, but multiple SQTS homologs were also found in single fungi of different groups^35^. The high number of different sesquiterpenes is generally mirrored in the *Alternaria* genome: A search of the *A. alternata* genome library (*Alternaria alternata*, SRC1lrK2f v1.0) revealed 30 putative terpene synthase genes (http://genome.jgi.doe.gov/pages/search-for-genes.jsf?organism=Altal1, accessed 04/11/2015).

VOC emissions from our *F. oxysporum* strain differed – except for acoradiene^23^ – from other strains described so far^22^,^23^,^24^, indicating that VOC profiles are strain-dependent in *F. oxysporum*. The lower number of compounds for *F. oxysporum* resulted in a much simpler correlation matrix compared to *A. alternata*. On the basis of this correlation matrix, we speculate that the same SQTS synthesized δ-elemene as the primary product along with several additional minor compounds. In accord with the lower number of detectable sesquiterpenes, only five putative terpene synthase genes were annotated in the *Fusarium oxysporum* genome (http://genome.jgi.doe.gov/pages/search-for-genes.jsf?organism=Fusox1, accessed 04/11/2015).

To translate the observed sesquiterpene emission pattern of both fungi to a molecular basis, future work will focus on the heterologous expression of the putative terpene synthase genes, genetic differences between strains^36^ and their functional biochemical characterization.

Our results clearly demonstrated highest sesquiterpene emissions in very young (3 days old) mycelia in both fungi. Contradictory to our findings, sesquiterpene emissions have been attributed to a later growth phase^20^,^28^. However, according to our literature survey, *Aspergillus versicolor* was the only truly indicative example for strongly increased sesquiterpene emissions with age, showing a 10^3^-fold increase between day 3 and 14 of incubation^37^. The early peak of emission in our study suggests that sesquiterpene formation is associated with rapid growth in *A. alternata* and *F. oxysporum*, at least on nutrient rich medium. For cultures on nutrient poor medium, we started VOC sampling when *A. alternata* cultures reached a size of 3 cm^2^, which occurred only after 14 days of incubation. Therefore, no information on emissions at early growth stages under nutrient poor cultivation conditions is available. Nonetheless, growth on nutrient poor media before day 14 was slower than during the following two weeks. If emissions are related to rapid growth as found under nutrient rich conditions, the growth pattern suggests no intensely increased emissions under nutrient poor conditions at an earlier time. However, this still needs to be studied further.

The general VOC pattern for *A. alternata* was stable during aging, except for an overly rapid decline in the alcohol emissions. This temporal emission pattern for small hydrocarbons is in accordance with other works. For example, in *Penicillium expansum*, C_8_ compounds were emitted mainly between an age of 3 and 9 days^20^. In contrast to the stable pattern found here, the VOC diversity of *Trichoderma atroviride* increased by 24 substances (three sesquiterpenes) in a comparison between 5- and 14-day-old cultures^38^. However, the effect of aging mycelia on sesquiterpene emissions has rarely been intentionally investigated and knowledge of the prevalence of sesquiterpene emissions in aging fungi remains fragmentary.

Emission quantities for *F. oxysporum* did not differ considerably between nutrient poor and rich media. Emissions from *A. alternata* from cultures of the same age strongly differed under rich and poor nutrient conditions, yet only when calculated for the entire plate but not with respect to the mycelium area. However, the physiological conditions between cultures of the same age under different nutrient conditions probably diverged strongly, which may explain the insignificant differences based on mycelial area. For example, *A. alternata* cultures on nutrient rich media had almost completed growing at day 14 and showed their final coloration, while *A. alternata* cultures on nutrient poor media at the same time covered only a tenth of the surface compared to their nutrient rich equivalents and were unpigmented. As mentioned above, cultures of *A. alternata* on nutrient poor medium likely never emitted VOCs in similar amounts as the cultures on nutrient rich medium. This suggests that the nutrient supply plays an essential role in sesquiterpene biosynthesis in *A. alternata*, although the mechanism remains unclear. A recent study on *Aspergillus fumigatus* showed that transcript levels of mevalonate kinase, one of the key enzymes in terpenoid biosynthesis, were decreased upon iron starvation, which resulted in a strong decline of sesquiterpene emissions^32^. The nutrient poor medium in our study did not contain iron, which may have been a reason for limited sesquiterpene biosynthesis.

Apart from the least abundant compounds, the volatile profile of *A. alternata* was the same under both nutrient conditions. Corresponding results were reported from *Penicillium polonicum*, *Aspergillus ustus* and *Periconia britannica* strains with similar sesquiterpene profiles when grown on malt extract, wallpaper, or plaster board^33^, or when several *Fusarium* spp. were cultivated on different substrates^21^.

Co-cultivation with shared headspace and separate growing space (split-plate) limited interactions between the fungi to their headspaces until the glass barrier was overgrown around day 10 after inoculation under nutrient rich conditions. However, the observed drop in total emissions from split-plates compared with the solitary cultivation of *A. alternata* was only significant at day 14, when possible effects of headspace interactions (e.g., change in emission profiles or amounts, adsorption of VOC by *F. oxysporum*) could not be separated from effects triggered by physical contact or exudates. However, during co-cultivation on split-plates, the headspace concentration of nearly all compounds was already lower very early at day 3 and 7 compared to the concentration found in solitary cultures of *A. alternata*. Because border effects were similar for both setups during this early stage, an active/passive adsorption of compounds in *F. oxysporum* mycelia is probable, especially because strains of *F. oxysporum* can transform terpenes^39^,^40^ and various fungi are known to grow with terpenes or similar compounds as a sole carbon source^41^. Another possibility is the reduced production of sesquiterpenes by *A. alternata* after recognition of *F. oxysporum* volatiles (i.e., δ-elemene). Although the exact mechanism must be elucidated in future studies, our experiment suggests a VOC-mediated interaction between the two fungal species.

The multivariate analysis clearly separated the sesquiterpene profiles of both fungi, suggesting that the profile might serve as a very good biomarker to discriminate between the tested strains of *A. alternata* and *F. oxysporum*. Such specific sesquiterpene profiles could also be used in ambient air to detect fungal growth. An earlier study^42^ concluded that sesquiterpene biomarkers may be the best approach to detect indoor fungal growth, after *Aspergillus*, *Chaetomium*, and *Epicoccum* were identified from water-damaged buildings via their sesquiterpene profiles. Examples of the use of different classes of VOCs as fungal biomarkers include screening for fungal causes of lung diseases^43^ and ecotyping^26^. The peculiar sesquiterpene profile of *A. alternata* is an ideal target for detecting those allergenic fungi in indoor environments. The specific emission pattern might be used to assemble sensitive VOC sensors (electronic nose) based on piezoelectric quartz crystals^44^, metal oxide semiconductors^45^, or metal-organic frameworks^46^. These sensors are capable of differentiating non-invasively and in real-time between different VOC profiles^44^ when coated and calibrated with specific compounds. Therefore, the identification of discriminant sesquiterpenes of *A. alternata* and *F. oxysporum* will pave the way for the development of future fungal sensors.

The available data on VOC emissions of different *F. oxysporum* strains^21^,^22^,^23^,^24^ suggests that sesquiterpenes are useful biomarkers on the sub-species level for this species, as has been proposed earlier^21^. The stability of sesquiterpene emissions by *A. alternata* under various conditions suggests that such emissions are especially good biomarkers for the growth of the species. However, intraspecific variability in *A. alternata* has not been assessed; therefore more *A. alternata* strains must be chemotyped. Because strains of *Alternaria* are known to produce different terpenoids (bi- and tricyloaleranenes) and other classes of toxins^14^, it may be speculated that a pathotype relation with sesquiterpene profiles could be established in a similar manner as has been demonstrated for *F. oxysporum*^21^.

This study showed the early, diverse and long-lasting emission of sesquiterpenes at a high rate and with a stable profile for the tested strain of *A. alternata*. If this observation holds true for different isolates of *A. alternata*, these properties make its emission profile useful for future chemotyping approaches in different fields, such as mycotoxin prevention or indoor health prediction. While this is also principally true for the investigated strain of *F. oxysporum*, low quantities of emissions might hamper such use.

Our novel findings concerning two frequently studied fungi highlight the importance of a thorough quantitative and qualitative (re-)evaluation of fungal VOC emissions of complex volatiles such as sesquiterpenes. Such fundamental analyses are necessary to provide a comprehensive knowledge base for mechanistic, ecological and applied research.

## Methods

### Culture media

A synthetic nutrient ‘poor’ and a complex nutrient ‘rich’ medium were prepared. The nutrient poor medium was based on synthetic nutrient poor agar and the nutrient rich medium on malt extract agar^47^. To minimize the volatile background, 15 g L^−1^ gelrite (SERVA electrophoresis, Heidelberg, Germany) were used for solidification instead of agar. The poor medium contained (L^−1^): KHPO_4_ 1 g, KNO_3_ 1 g, MgSO_4_ × 7 H_2_O 0.5 g, KCl 0.5 g, glucose (D+) 0.2 g, sucrose 0.2 g, ZnSO_4_ × 7 H_2_O 0.01 g, CuSO_4_ × 5 H_2_O 0.005 g; the rich medium contained (L^−1^): malt extract 20 g, ZnSO_4_ × 7 H_2_O 0.01 g, CuSO_4_ × 5 H_2_O 0.005 g. The media were poured (20 ml plate^−1^) into sterile degreased glass petri-dishes without division (direct confrontation) or with a glass-barrier of 7 mm height dividing the plate into two equal halves (split-plate). The split-plates ensured that only VOC-mediated interactions were possible during early growth, while on direct confrontations exudates into the medium and early exploratory hyphae were additional routes for interactions. After solidification, each dish of nutrient poor medium was provided with four cellulose filters (30 mm, No. 1001-329, Whatman GE, Dassel, Germany) that served as a polymer carbon source and a supporting matrix for mycelia to approximate the situation in a withering plant. Sterile culture plates were stored at least one week at 4 °C in odorless roasting tubes (Toppits Cofresco, Minden, Germany) before use.

### Strains and cultivation

The experimental plates were inoculated with 3 × 50 conidial spores from spore-solutions made from *Alternaria alternata* (Fr.) Keissler (DSMZ 62006) or *Fusarium oxysporum* f. *aechmeae* (Fr.) Schltdl. (DSMZ 62297) cultures grown as recommended^47^. For the rich medium, the spores were applied in three droplets onto one half of the plate and approximately 1 cm from the rim with equal spacing between the droplets. For the poor medium, two droplets were applied on half of the cellulose-filter next to the rim of the dish and one droplet was placed in between the filters. The plates were sealed with Parafilm M (Bemis, Oshkosh, WI, USA) to preserve humidity (<0.5% loss of weight in a 30 days incubation pre-test) and incubated at 20 °C in the dark.

On both media, five growth setups were tested in five replicate plates each: (1) plates with sterile media for sampling the VOC-background, (2) *A. alternata* on one half of a split-plate with the second half containing sterile medium (‘solitary *A. alternata*’), (3) *F. oxysporum* on a half of a split-plate with the second half containing sterile medium (‘solitary *F. oxysporum*’), (4) both fungi on different halves of a shared split-plate, (5) both fungi on opposite sides of a barrier-less plate (‘direct confrontation’). For each growth condition, three extra replicates were incubated under exactly the same conditions except for absence of VOC sampling, to monitor possible changes in the growth dynamics compared to the sampled plates because sampling is a potential growth disturbance (e.g., short exposure to light and lifting of the lid).

### Collection of VOCs and the sampling scheme

VOCs were collected from the glass Petri dishes by head-space sorptive extraction using the SBSE method based on non-polar, polydimethylsiloxane coated stir bars (Twister, film thickness 0.5 mm, Gerstel, Mülheim an der Ruhr, Germany) as recently described^26^, with the following modification - magnetic discs were fixed to the dishes in the center of each lid’s outer surface. This guaranteed a central positioning of the magnetic sorptive stir bars in the air-space of the plates and rapid, contamination-free handling (Supplementary Fig. S7). The collection time was 48 hours for cultures on poor and 20 hours for cultures on rich medium.

Sampling began when the slower growing fungus covered approximately 1 to 3 cm^2^ of the plate surface. Four time-points were sampled from nutrient poor and five from nutrient rich medium. Time-points for sampling from nutrient poor plates were days 14, 21, 28, and day 35 after inoculation, and the time-points for nutrient rich plates were days 3, 7, 14, 21, and day 28 (each date was the beginning of a VOC collection, e.g., sampling for day 3 started 72 hours after inoculation).

### Analyses of VOCs

In our approach, as in recent work^26^, VOC samples were analyzed using a thermo-desorption unit (TDU, Gerstel) coupled to a GC-MS (GC type: 7890A, MS type: 5975C inert XL MSD with a triple axis detector, both from Agilent Technologies (Palo Alto, CA, USA) using a 5% phenyl 95% dimethyl arylene siloxane capillary column (60 m × 250 μm × 0.25 μm DB-5MS + 10 m DG, Agilent Technologies). The TDU-GC-MS was run as described previously^48^,^49^. Calibration was achieved by injecting pure standard mixtures dissolved in hexane at seven different concentrations ranging from ~20 to 800 pmol μl^−1^. Each mixture was made independently in triplicate for each concentration and measured in duplicate. The resulting MS signal responses were found to be linear with an increasing standard concentration with *R*^*2*^ > 0.99. Non-isothermal Kovats retention indices were calculated according to generally accepted standards^50^, based on chromatography retention times of a saturated alkane mixture (C_7_ – C_40_; Sigma-Aldrich, Taufkirchen, Germany) and other alkanes (<C_7_) occurring in the chromatogram background.

Limits of detection (LOD) were set to twice σ, and the limit of quantification (LOQ) to 10-folder of LOD. Emission rates were calculated on a fungal mycelium area (pmol cm^−2^ h^−1^) or plate (pmol h^−1^) bases.

### Statistical analysis

Relationships between VOC emissions from *A. alternata* and *F. oxysporum* growing solitary or in co-cultures in nutrient rich medium were analyzed using principal component analysis (PCA) and orthogonal partial least squares discriminant analysis (OPLS) (SIMCA-P v13, Umetrics, Umeå, Sweden). For this, day 7 was chosen because all VOCs were detected and the highest plate-based emission rates were measured at this date. Using plate-based emission rates allowed the inclusion of data for the co-cultivation setups. Established procedures to analyses and evaluate MS data were followed in a similar manner as previously described^48^,^51^,^52^,^53^,^54^,^55^,^56^. Prior to analysis, all x-variables were logarithmically (log10) transformed, centered, and each type of data was scaled block-wise with 1 sd^−1^. Calculated significant principal components were validated using ‘full cross-validation’, with 95% confidence level on parameters and 7 as number of cross-validation groups. The prediction ability of the OPLS model to discriminate *A. alternata* and *F. oxysporum* was evaluated using VOC data collected at day 3, 7, 14.

Spearman correlation-matrices for the emissions of the volatile substances to each other were calculated in the R programming environment^57^ using algorithms of the Hmisc package^58^ (v3.10-1). Hierarchical clustering (single linkage) of the results was performed with the corrplot package^59^ (v0.73).

## Additional Information

**How to cite this article**: Weikl, F. *et al.* Sesquiterpene emissions from *Alternaria alternata* and *Fusarium oxysporum*: Effects of age, nutrient availability, and co-cultivation. *Sci. Rep.* **6**, 22152; doi: 10.1038/srep22152 (2016).

## Supplementary Material

Supplementary Information

## Figures and Tables

**Table 1:** VOC analysis using stir bar sorptive extraction (SBSE) and GC-MS. Means of plate based VOC emission rates ± s.e. (n = 5) from 7 days old *Alternaria alternata* and *Fusarium oxysporum* co-cultures (confrontation plates) growing on nutrient rich medium (malt extract gelrite).

Compounds	RT(min)	Kovats’RI	CAS	LOD(pmol plate^−1^ h^−1^)	emission(pmol plate^−1^ h^−1^)
alcohols					
Isobutanol	6.827	619	78-83-1	<0.01	4.88 ± 0.97
2-Methyl-1-butanol	8.695	724	137-32-6	<0.01	8.44 ± 1.92
sesquiterpenes					
δ-Elemene	32.864	1335	20307-84-0	<0.01	13.39 ± 1.03
β-Elemene	35.067	1391	515-13-9	0.05	4.98 ± 0.34
Di-epi-α-Cedrene	35.822	1410	50894-66-1	<0.01	11.67 ± 0.62
α-Cedrene	36.422	1427	11028-42-5	<0.01	14.3 ± 0.7
β-Cedrene	36.861	1439	546-28-1	<0.01	421.31 ± 20.92
Thujopsene	37.252	1449	470-40-6	<0.01	206.11 ± 18.37
(E)-β-Farnesene^a^	37.355	1452	18794-84-8	0.02	6.18 ± 0.34
β-Acoradiene	38.083	1472	24048-44-0	<0.01	24.36 ± 1.29
unknown SQT^b^ #1	38.304	1478	n.a.	0.06	92.5 ± 5.73
α-Himachalene	38.44	1482	3853-83-6	<0.01	22.49 ± 1.52
unknown SQT #2	38.684	1488	n.a.	<0.01	2.38 ± 0.49
β-Chamigrene	38.92	1495	18431-82-8	<0.01	16.91 ± 0.78
allo-Aromadendrene	39.138	1501	25246-27-9	0.14	14.65 ± 0.94
γ-Curcumene	39.322	1508	28976-68-3	<0.01	15.08 ± 2.06
Germacrene D	39.559	1517	23986-74-5	<0.01	20.66 ± 1.22
Eremophilene	39.86	1529	10219-75-7	<0.01	6.35 ± 0.43
α-Chamigrene	40.257	1545	19912-83-5	<0.01	6.78 ± 0.41
γ-Cadinene	40.323	1548	1460-97-5	<0.01	10.76 ± 0.58
unknown SQT #3	41.478	1593	n.a.	0.03	2.76 ± 0.22
oxygenated sesquiterpenes					
Thujopsane-2β-ol + unknown SQT #4^c^	41.761	1606	150737-93-2n.a.	<0.01	33.07 ± 3.95
Cedren-13-ol, 8-	42.399	1641	18319-35-2	<0.01	1.35 ± 0.14
Widdrol	42	1619	6892-80-4	<0.01	3.47 ± 0.31
10-epi-γ-Eudesmol	42.247	1632	15051-81-7	<0.01	3.14 ± 0.26

Emission rates for further time-points and the other experiments are shown in Supplementary Tables S1–4.

^a^verified by authentical standards, otherwise tentatively identified.

^b^SQT: sesquiterpene.

^c^co-eluted peaks.

**Table 2:**
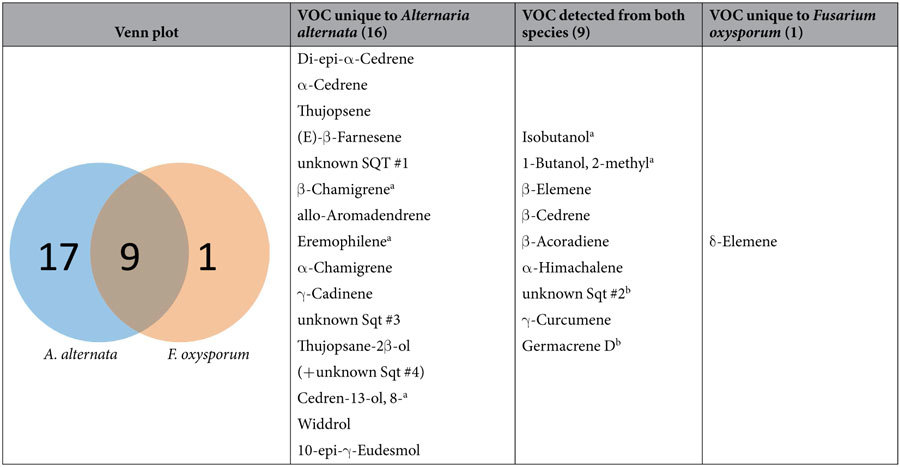
Fungal origin of volatile organic compounds (VOC) (above the limit of quantification (pmol plate^−1^ h^−1^)) collected from solitary cultures of *Alternaria alternata* and *Fusarium oxysporum* grown on nutrient rich medium (malt extract gelrite).

^a^absent on nutrient poor medium, ^b^on nutrient poor medium only detected for *A. alternata*.
