# Supplementary material for: Sesquiterpene emissions from Alternaria alternata and Fusarium oxysporum: Effects of age, nutrient availability, and co-cultivation
Source: Sci Rep. 2016 Feb 26;6:22152. doi: 10.1038/srep22152 (PMC4768142; doi:10.1038/srep22152)
Supplement: Supplementary Information [file srep22152-s1.pdf]

Supplementary Materials

Sesquiterpene emissions from *Alternaria alternata* and *Fusarium oxysporum*:

Effects of age, nutrient availability, and co-cultivation

Fabian Weikl, Andrea Ghirardo, Jörg-Peter Schnitzler, Karin Pritsch

Table of Contents:

Supplementary Figures

Figure S1
Figure S2
Figure S3
Figure S4
Figure S5
Figure S6
Figure S7

Supplementary Tables

Table S1
Table S2
Table S3
Table S4

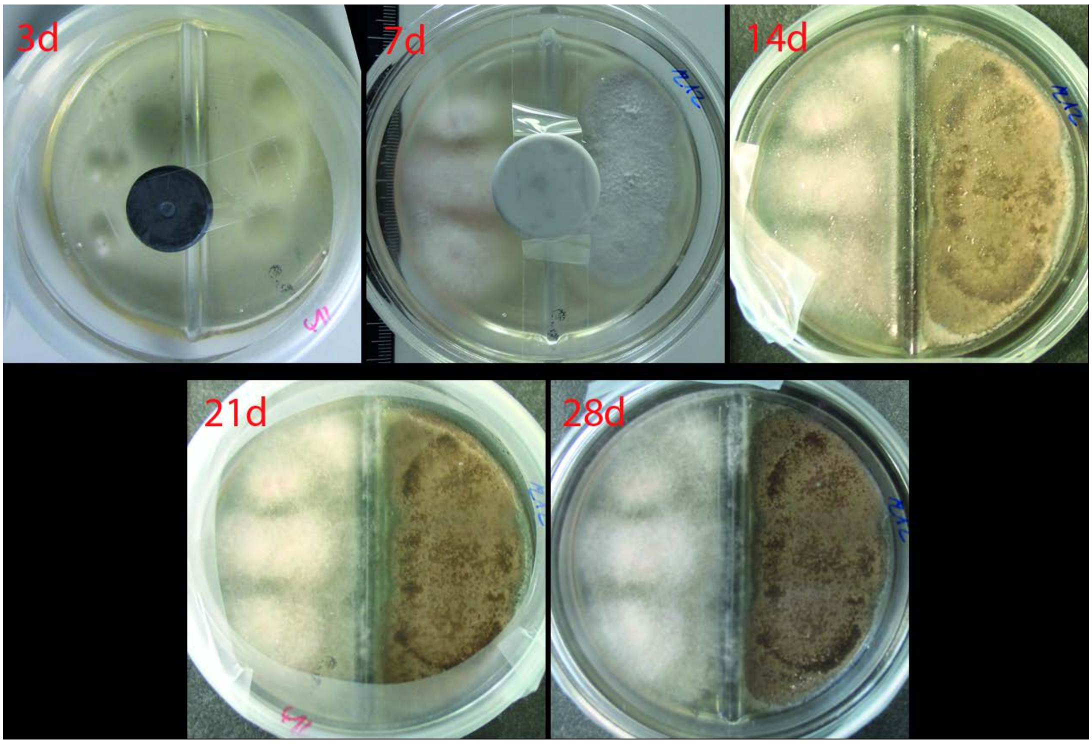

Figure S1 Time series (dates of VOC samplings) on the morphology of a shared split-plate with nutrient rich conditions. Left halves: *Fusarium oxysporum*, right halves: *Alternaria alternata*. d: days after inoculation.

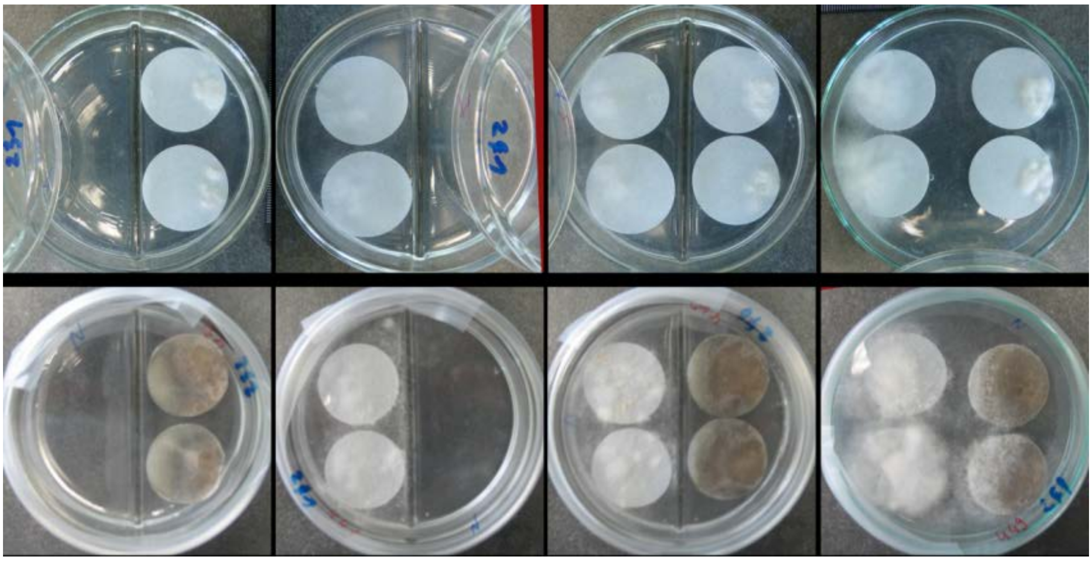

Figure S2 Culture morphology under nutrient poor conditions. Upper panel: at the first VOC sampling (day 14 after inoculation); lower panel: at the last VOC-sampling (day 35). From left to right: *Alternaria alternata* solitary, *Fusarium oxysporum* solitary, shared split-plate (left half *F. oxysporum*, right half *A. alternata*), direct confrontation (left half *F. oxysporum*, right half *A. alternata*).

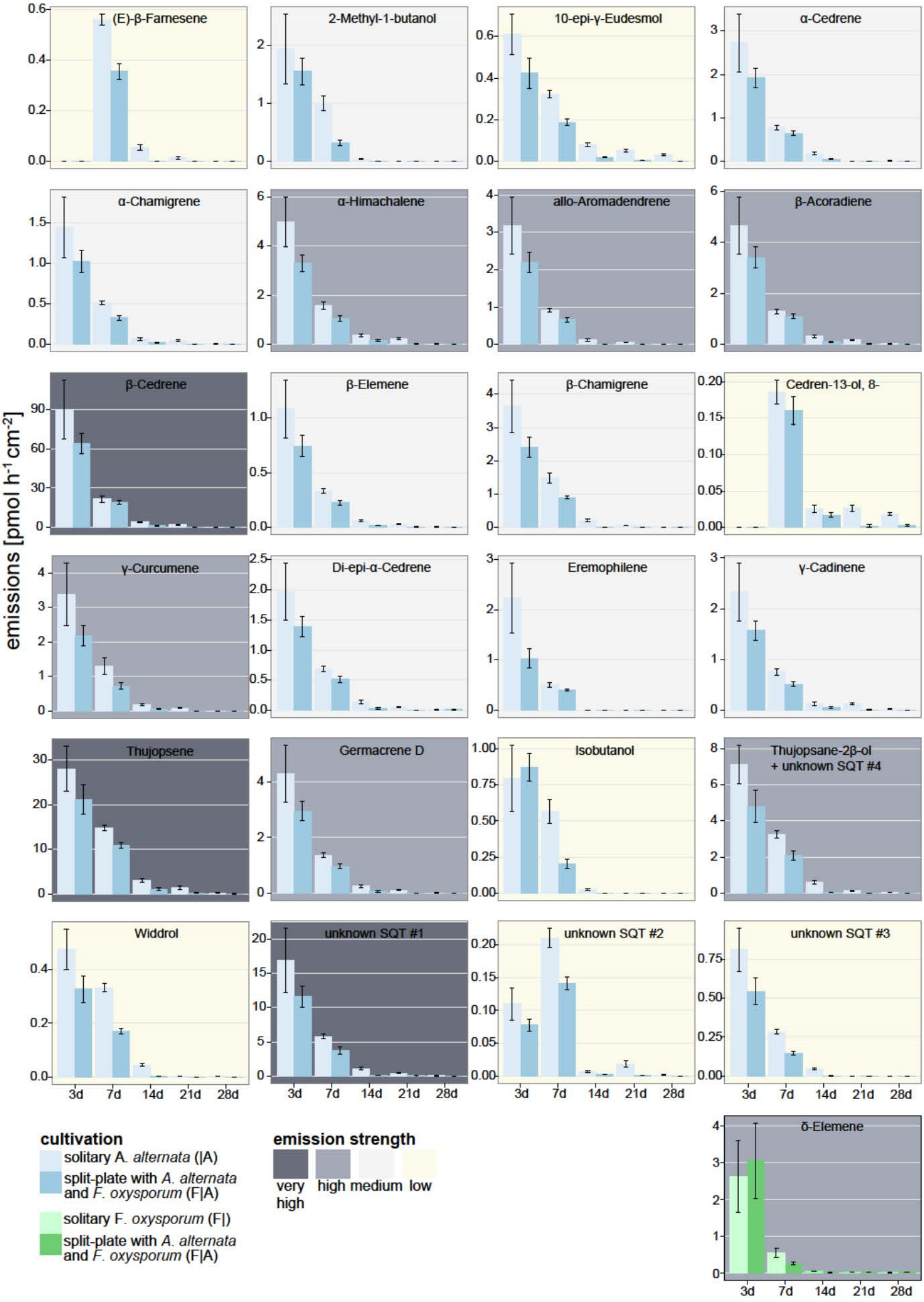

Figure S3 Comparisons between solitarily grown *Alternaria alternata* (solitary *Fusarium oxysporum* in the case of δ-elemene) and split-plates with *A. alternata* and *F. oxysporum* on nutrient rich medium. Emission rates of each compound calculated based on *A. alternata* mycelium area, except for δ-elemene, for which emission rates were calculated based on *F. oxysporum* mycelium area; error bars: s.e. (n = 5).

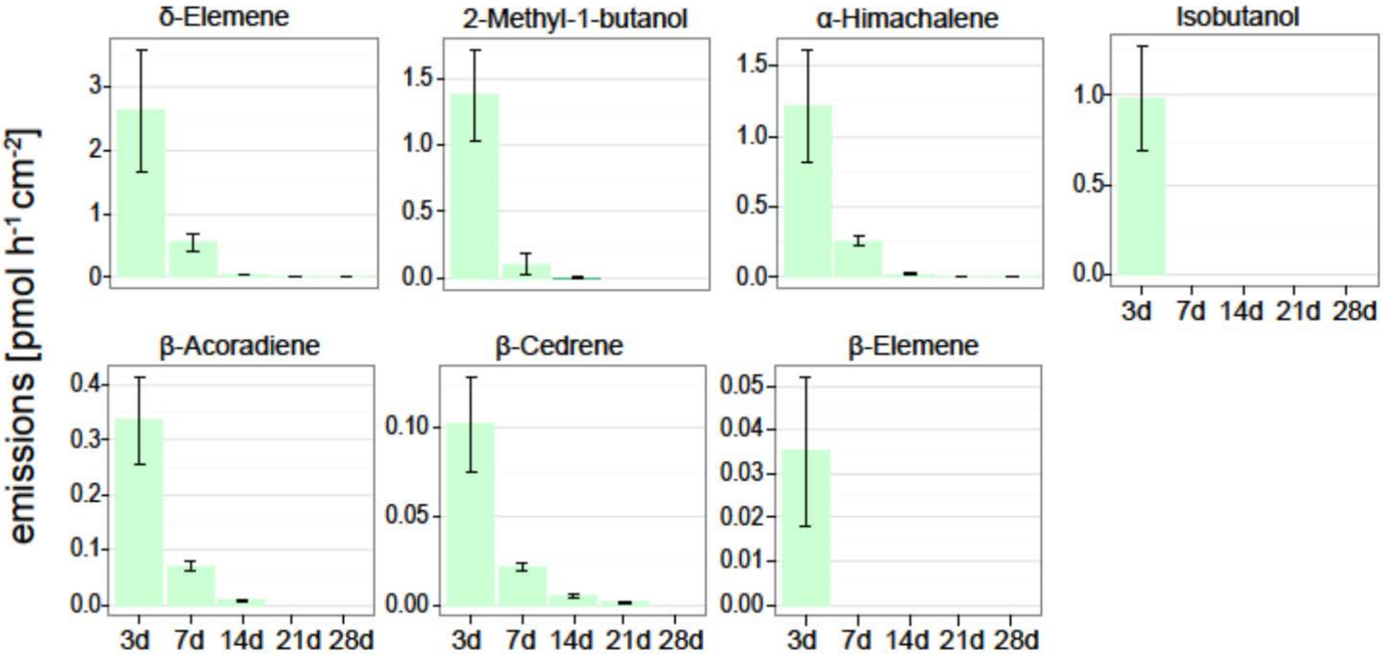

Figure S4 Emission rates of solitarily grown *Fusarium oxysporum* on nutrient rich medium. Emission rates of each compound calculated based on *F. oxysporum* mycelium area; error bars: s.e. (n = 5). The substances emitted at rates lower than 0.01 pmol cm^{-2} h^{-1} (γ-curcurmene, germacrene-D, and the unknown SQT #2) are not displayed (cf. Supplementary Table S3).

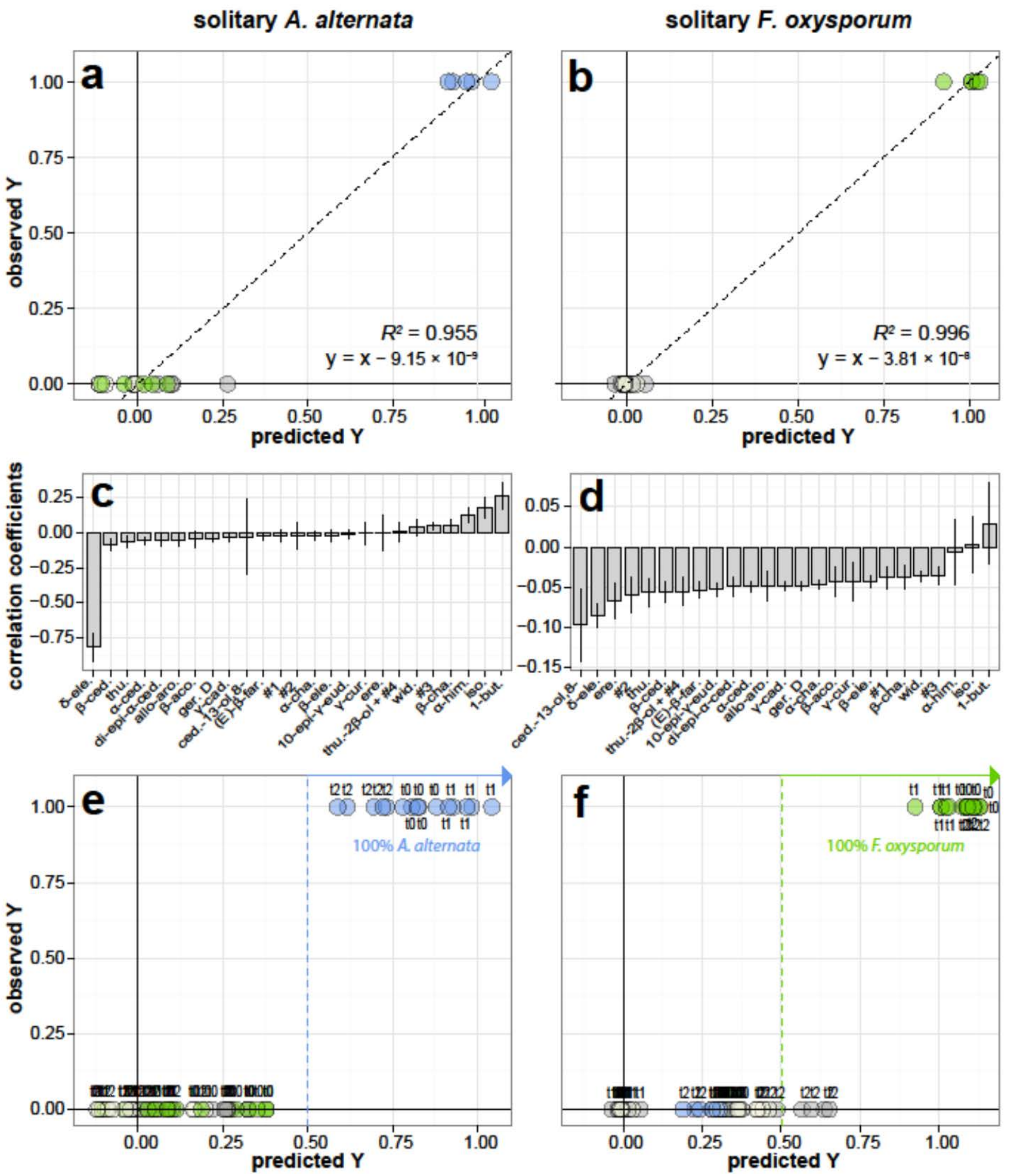

Figure S5 Observed versus predicted Y-values of orthogonal partial least square (OPLS) analysis referred to *Alternaria alternata* (a) and *Fusarium oxysporum* (b) modeled using VOC profile data from 7 days old culture and growing with nutrient rich media, and corresponding correlation coefficient plots (c, d); prediction plots of VOC profiles from day 3,7,14 (nutrient rich) (e, f): predicted Y-values > 0.5 are classified as *A. alternata* in (e) and *F.oxysporum* in (f). Colored dots: blue: solitarily grown *A. alternata*, green: solitarily grown *F. oxysporum*, grey: split-plates with both fungi, beige: direct confrontations. Error bars: confidence intervals based on jack-knifing uncertainty method. Regression lines: goodness of fit. OPLS model fitness: R^2(X) = 98.6%, R^2(Y) = 100%, r^2 = 76.8%, Q^2(cum) = 70.1% using 3 predictive components . RMSEE: *A. alternata* = 0.102; *F. oxysporum* = 0.029; RMSEcv: *A. alternata* = 0.101; *F. oxysporum* =0.031. P-values of cross-validated ANOVA: *A. alternata* (solitary), $P < 2 \times 10^{-7}$; *F. oxysporum* $P < 4 \times 10^{-14}$. Partly overplotted text (e, f): t1 (day 3 VOC sampling), t1 (day 7), t2 (day 14).

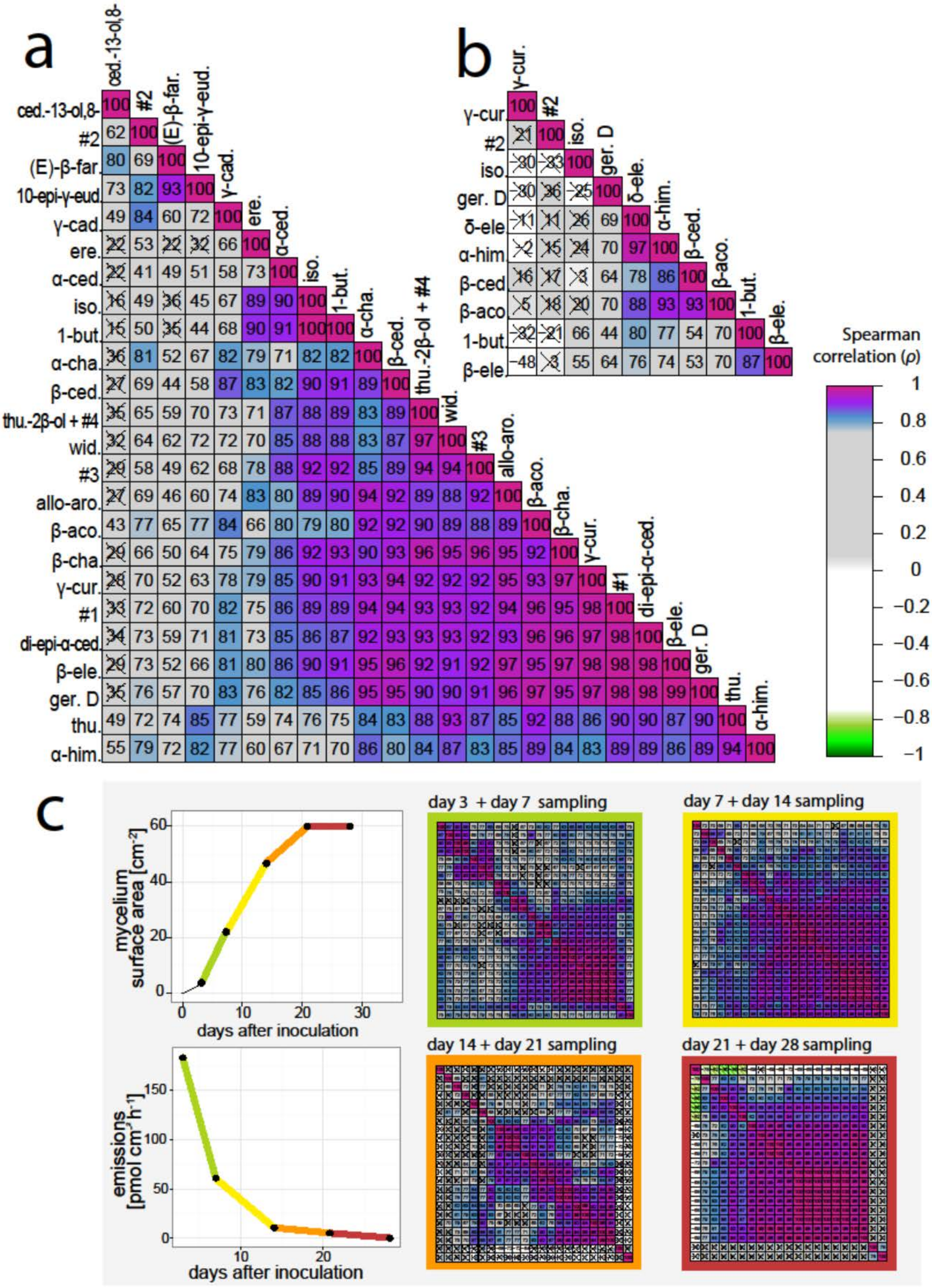

Figure S6 Correlation matrices for emissions form solitary cultures on nutrient rich medium. (a,b): Lower triangle correlation matrices including data from all time-points and replicates. Compounds ordered by hierarchical clustering (single linkage). Numbers: Spearman correlation coefficient (ρ) expressed as percentage; X: correlation insignificant ($P > 0.05$). (a) *Alternaria alternata,* (b) *Fusarium oxysporum*. (c) *A. alternata*: correlation matrices including data of all replicates of two consecutive time-points each. Matrices were prepared as for (a), order of compounds differs since clustering was used. Colored frames around the matrices correspond to the respective times covered in the graphs to the left (emission rates and mycelium surface areas).

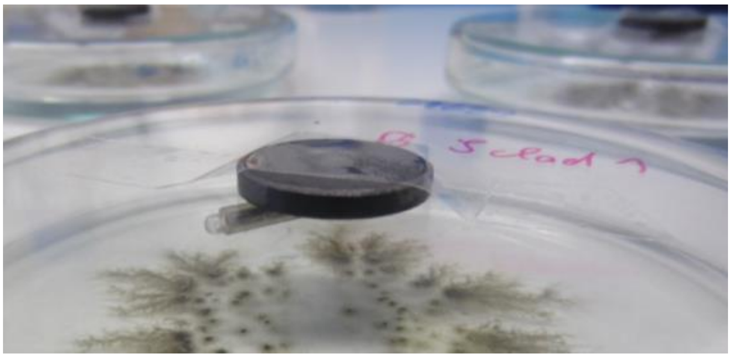

Figure S7 Example of a PDMS-twister (polydimethylsiloxane coated stir bar) attached to the inside of a glass culture-dish's lid and held in place by a magnet on the outside of the dish (pre-test with a *Cladosporium* isolate).

Table S1 VOC mission rate per plate (pmol plate^{-1} h^{-1}) for all sampling dates (days post inoculation) of nutrient rich and nutrient poor cultures of solitary fungi; s.e.: standard error (n = 5); nd: not detectable.

***Alternaria alternata* solitary |A**

	**nutrient rich (malt extract gelrite)**										**nutrient poor (synth. nutrient poor gelrite)**							
Compounds	3 days	s.e.	7 days	s.e.	14 days	s.e.	21 days	s.e.	28 days	s.e.	14 days	s.e.	21 days	s.e.	28 days	s.e.	35 days	s.e.
Isobutanol	2.2	0.13	11.9	1.9	1.14	0.24	nd	nd	nd	nd	nd	nd	nd	nd	nd	nd	nd	nd
2-Methyl-1-butanol	5.21	0.37	21.1	2.5	1.89	0.39	nd	nd	nd	nd	nd	nd	nd	nd	nd	nd	nd	nd
β-Elemene	3.0	0.20	6.92	0.46	2.87	0.52	1.70	0.28	0.260	0.10	0.143	0.01	0.046	<0.01	nd	nd	0.028	<0.01
Di-epi-α-cedrene	5.44	0.34	14.3	0.8	6.44	1.72	3.17	0.62	0.516	0.14	0.237	0.03	0.064	0.01	nd	nd	0.041	<0.01
α-Cedrene	7.5	0.44	16.3	0.6	8.73	1.33	nd	nd	0.789	0.32	nd	nd	0.056	0.02	nd	nd	0.079	<0.01
β-Cedrene	248.5	15.6	446.5	39.6	189.7	20.2	127.7	23.1	10.9	5.4	9.21	1.15	2.26	0.54	0.185	0.07	1.55	0.02
Thujopsene	82.2	8.1	308.1	6.5	144.0	20.1	87.3	24.6	10.6	7.0	3.90	0.31	4.18	0.54	1.45	0.16	2.66	0.17
δ-Elemene	nd	nd	nd	nd	nd	nd	nd	nd	nd	nd	nd	nd	nd	nd	nd	nd	nd	nd
(E)-β-Farnesene	nd	nd	11.8	0.4	2.57	0.55	0.861	0.26	0.045	0.01	0.211	0.02	0.056	0.02	0.035	<0.01	0.016	<0.01
β-acoradiene	12.9	0.8	26.8	1.1	15.0	2.6	10.4	1.73	2.16	0.72	0.844	0.15	0.236	0.03	nd	nd	nd	nd
α-Himachalene	13.6	2.0	33.0	3.3	17.6	3.0	14.2	2.57	1.21	0.57	1.25	0.14	0.291	0.08	nd	nd	0.234	<0.01
β-Chamigrene	10.8	0.7	31.0	2.5	9.60	1.66	3.41	0.54	nd	nd	nd	nd	nd	nd	nd	nd	nd	nd
unknown SQT #2	0.308	0.02	4.4	0.3	0.330	0.06	1.10	0.27	0.083	0.05	0.093	0.01	0.004	<0.01	0.015	<0.01	0.024	<0.01
unknown SQT #1	43.7	4.3	121.0	7.0	50.9	10.2	25.3	3.5	3.42	1.30	0.329	0.03	0.623	0.16	nd	nd	nd	nd
allo-Aromadendrene	8.8	0.52	19.2	1.3	5.77	1.58	3.35	0.60	0.229	0.11	0.276	0.11	nd	nd	nd	nd	nd	nd
γ-Curcumene	9.30	0.73	27.2	4.4	9.07	1.39	5.09	1.05	nd	nd	0.353	0.08	0.241	0.01	0.122	0.02	nd	nd
Germacrene D	11.9	0.7	28.5	1.8	11.9	2.2	6.83	1.53	0.553	0.34	0.505	0.06	0.163	0.04	0.031	<0.01	0.114	<0.01
Eremophilene	5.5	0.63	10.8	1.4	nd	nd	nd	nd	nd	nd	nd	nd	nd	nd	nd	nd	nd	nd
α-Chamigrene	4.0	0.18	10.8	0.5	3.10	0.62	2.92	0.46	0.607	0.24	0.123	0.01	0.039	<0.01	0.005	<0.01	0.032	<0.01
γ-cadinene	6.42	0.41	15.9	1.5	6.22	1.58	7.34	1.15	1.47	0.57	0.293	0.04	0.094	0.02	nd	nd	0.072	<0.01
unknown SQT #3	2.4	0.17	5.96	0.32	2.26	0.26	nd	nd	0.064	<0.01	0.160	0.01	0.046	<0.01	nd	nd	0.058	<0.01
Thujopsane-2β-ol +unknown SQT #4	23.4	3.0	67.6	2.6	28.9	4.8	8.35	1.23	2.01	0.24	nd	nd	nd	nd	1.04	0.04	1.57	0.22
Widdrol	1.44	0.15	7.0	0.35	2.11	0.30	0.179	0.02	0.048	<0.01	nd	nd	nd	nd	0.130	0.02	0.043	<0.01
10-epi-γ-Eudesmol	1.86	0.18	6.73	0.26	3.80	0.39	3.17	0.33	1.85	0.23	0.233	0.02	0.151	0.01	0.095	0.01	0.155	0.01
Cedren-13-ol, 8-	nd	nd	3.91	0.36	1.17	0.21	1.56	0.24	1.11	0.09	nd	nd	nd	nd	nd	nd	nd	nd

***Fusarium oxysporum* solitary F|**

Compounds	3 days	s.e.	7 days	s.e.	14 days	s.e.	21 days	s.e.	28 days	s.e.	14 days	s.e.	21 days	s.e.	28 days	s.e.	35 days	s.e.
Isobutanol	0.987	0.16	nd	nd	nd	nd	nd	nd	nd	nd	0.004	<0.01	nd	nd	nd	nd	nd	nd
2-Methyl-1-butanol	1.43	0.24	1.17	0.78	0.093	0.04	nd	nd	nd	nd	nd	nd	nd	nd	nd	nd	nd	nd
β-Elemene	0.028	<0.01	nd	nd	nd	nd	nd	nd	nd	nd	0.004	<0.01	0.001	<0.01	nd	nd	nd	nd
β-Cedrene	0.107	0.02	0.361	0.05	0.203	0.06	0.073	0.03	nd	nd	nd	nd	nd	nd	nd	nd	nd	nd
δ-Elemene	2.44	0.17	8.40	0.66	2.01	0.38	1.01	0.25	0.587	0.14	0.552	0.15	nd	nd	0.127	0.05	nd	nd
β-acoradiene	0.360	0.06	1.16	0.16	0.316	0.08	0.109	0.05	nd	nd	0.298	0.08	0.039	<0.01	nd	nd	nd	nd
α-Himachalene	1.17	0.10	4.09	0.28	1.078	0.18	0.54	0.14	0.291	0.08	0.279	0.07	0.137	0.02	nd	nd	0.044	<0.01
unknown SQT #2	nd	nd	0.009	<0.01	nd	nd	0.010	<0.01	nd	nd	nd	nd	nd	nd	nd	nd	nd	nd
γ-Curcumene	nd	nd	nd	nd	0.105	0.03	0.111	0.05	nd	nd	0.011	0.01	nd	nd	0.060	0.02	nd	nd
Germacrene D	nd	nd	0.064	<0.01	nd	nd	nd	nd	nd	nd	nd	nd	nd	nd	nd	nd	nd	nd

Table S2 VOC mission rate per plate (pmol plate^{-1} h^{-1}) for all sampling dates (days post inoculation) of nutrient rich and nutrient poor co-cultivations; s.e.: standard error (n = 5); nd: not detectable.

split-plate F|A

	**nutrient rich (malt extract gelrite)**										**nutrient poor (synth. nutrient poor gelrite)**							
	3		7		14		21		28		14		21		28		35	
Compounds	days	s.e.	days	s.e.	days	s.e.	days	s.e.	days	s.e.	days	s.e.	days	s.e.	days	s.e.	days	s.e.
Isobutanol	3.01	0.19	4.17	0.70	nd	nd	nd	nd	nd	nd	nd	nd	nd	nd	nd	nd	nd	nd
2-Methyl-1-butanol	5.29	0.55	6.65	1.00	nd	nd	nd	nd	nd	nd	nd	nd	nd	nd	nd	nd	nd	nd
β-Elemene	2.53	0.17	4.60	0.43	0.472	0.07	0.109	0.04	0.043	0.02	0.116	0.02	0.031	<0.01	nd	nd	nd	nd
Di-epi-α-cedrene	4.75	0.25	10.5	1.2	0.822	0.25	nd	nd	0.253	0.07	0.181	0.03	0.034	<0.01	nd	nd	0.030	<0.01
α-Cedrene	6.58	0.31	13.2	1.2	1.43	0.27	0.301	0.10	0.088	0.05	nd	nd	0.053	<0.01	nd	nd	0.021	<0.01
β-Cedrene	218.2	11.5	390.1	32.6	35.6	9.1	3.69	1.76	0.769	0.43	6.71	1.16	1.11	0.07	nd	nd	nd	nd
Thujopsene	72.7	9.3	219.3	11.4	28.7	9.1	4.52	2.93	0.960	0.52	2.66	0.36	1.36	0.08	0.099	0.02	0.011	0.01
δ-Elemene	2.06	0.15	4.81	0.63	0.236	0.11	0.584	0.09	0.329	0.04	0.483	0.05	nd	nd	0.430	0.07	nd	nd
(E)-β-Farnesene	nd	nd	7.29	0.74	0.039	<0.01	nd	nd	0.015	<0.01	0.171	0.04	0.022	<0.01	nd	nd	nd	nd
β-acoradiene	11.6	0.6	22.4	2.0	2.694	0.54	0.538	0.19	0.410	0.25	0.817	0.21	0.124	0.03	nd	nd	nd	nd
α-Himachalene	12.0	2.1	21.3	2.3	4.30	1.51	0.577	0.18	0.235	0.05	1.12	0.14	0.175	<0.01	0.247	0.02	0.084	<0.01
β-Chamigrene	8.23	0.50	18.4	1.5	nd	nd	nd	nd	nd	nd	nd	nd	nd	nd	nd	nd	nd	nd
unknown SQT #2	0.27	0.01	2.88	0.27	0.053	0.01	0.027	0.01	0.009	<0.01	0.069	<0.01	0.002	<0.01	0.015	<0.01	nd	nd
unknown SQT #1	39.7	2.5	76.5	11.4	3.10	0.29	1.42	0.21	nd	nd	0.862	0.53	0.233	0.02	nd	nd	nd	nd
allo-Aromadendrene	7.51	0.39	13.4	1.2	0.382	0.11	nd	nd	nd	nd	0.297	0.14	nd	nd	nd	nd	nd	nd
γ-Curcumene	7.57	0.75	14.6	1.6	1.53	0.51	0.099	0.07	nd	nd	0.508	0.11	nd	nd	0.141	0.02	nd	nd
Germacrene D	10.1	0.5	19.7	1.8	1.69	0.68	0.135	0.08	0.034	0.01	0.371	0.06	0.064	<0.01	0.002	<0.01	nd	nd
Eremophilene	3.58	0.70	8.3	0.4	nd	nd	nd	nd	nd	nd	nd	nd	nd	nd	nd	nd	nd	nd
α-Chamigrene	3.50	0.21	6.75	0.65	0.584	0.14	0.091	0.03	0.019	0.02	0.114	0.03	0.023	<0.01	nd	nd	nd	nd
γ-cadinene	5.39	0.29	10.5	1.0	1.47	0.35	0.207	0.08	0.073	0.03	0.233	0.04	0.053	<0.01	nd	nd	0.002	<0.01
unknown SQT #3	1.87	0.24	2.95	0.32	0.045	0.02	nd	nd	nd	nd	nd	nd	0.023	<0.01	nd	nd	nd	nd
Thujopsane-2β-ol +unknown SQT #4	16.3	2.0	42.8	5.9	1.35	0.31	nd	nd	nd	nd	nd	nd	nd	nd	nd	nd	nd	nd
Widdrol	1.14	0.15	3.50	0.19	0.035	0.01	nd	nd	nd	nd	nd	nd	nd	nd	nd	nd	nd	nd
10-epi-γ-Eudesmol	1.45	0.21	3.87	0.41	0.568	0.12	0.148	0.02	0.044	<0.01	nd	nd	0.058	<0.01	0.007	<0.01	nd	nd
Cedren-13-ol, 8-	nd	nd	3.27	0.42	0.459	0.10	0.065	0.05	0.082	0.04	nd	nd	nd	nd	nd	nd	nd	nd

direct confrontation FA

	3		7		14		21		28		14		21		28		35	
Compounds	days	s.e.	days	s.e.	days	s.e.	days	s.e.	days	s.e.	days	s.e.	days	s.e.	days	s.e.	days	s.e.
Isobutanol	2.28	0.18	4.88	0.97	nd	nd	nd	nd	nd	nd	nd	nd	nd	nd	nd	nd	nd	nd
2-Methyl-1-butanol	4.07	0.38	8.44	1.92	nd	nd	nd	nd	nd	nd	nd	nd	nd	nd	nd	nd	nd	nd
β-Elemene	1.82	0.22	4.98	0.34	1.38	0.11	0.369	0.03	0.094	0.03	0.610	0.18	0.046	<0.01	nd	nd	nd	nd
Di-epi-α-cedrene	3.31	0.42	11.7	0.6	3.45	0.34	0.462	0.07	1.16	0.49	1.26	0.41	0.059	0.01	nd	nd	0.041	0.02
α-Cedrene	4.68	0.57	14.3	0.7	4.61	0.47	1.00	0.13	0.209	0.09	nd	nd	0.092	0.01	nd	nd	0.030	<0.01
β-Cedrene	152.5	19.6	421.3	21.0	127.5	12.6	15.9	3.3	2.16	1.14	53.6	18.0	2.26	0.34	nd	nd	nd	nd
Thujopsene	38.73	3.9	206.1	18.4	86.3	6.7	12.5	2.0	1.64	0.89	5.94	1.81	1.09	0.09	nd	nd	0.009	<0.01
δ-Elemene	3.83	0.33	13.4	1.03	0.562	0.08	0.630	0.11	0.282	0.08	1.18	0.30	nd	nd	0.482	0.13	nd	nd
(E)-β-Farnesene	1.01	0.10	6.18	0.34	nd	nd	nd	nd	0.030	0.01	0.967	0.37	0.036	<0.01	0.013	<0.01	nd	nd
β-acoradiene	8.23	1.02	24.4	1.3	7.67	0.75	1.34	0.24	0.805	0.34	3.50	0.92	0.203	0.04	nd	nd	nd	nd
α-Himachalene	12.66	2.1	22.5	1.5	14.6	1.8	1.45	0.32	0.538	0.14	6.67	1.86	0.295	0.03	0.583	0.17	0.148	0.02
β-Chamigrene	4.61	0.37	16.9	0.8	nd	nd	nd	nd	nd	nd	nd	nd	nd	nd	nd	nd	nd	nd
unknown SQT #2	0.184	0.02	2.38	0.49	0.167	0.02	0.086	0.02	0.011	<0.01	0.440	0.13	0.002	<0.01	0.014	<0.01	nd	nd
unknown SQT #1	27.5	2.1	92.5	5.7	11.5	3.8	3.02	0.35	0.822	0.31	0.959	0.34	0.508	0.07	nd	nd	nd	nd
allo-Aromadendrene	5.25	0.63	14.7	0.9	3.15	0.41	nd	nd	nd	nd	1.87	0.58	nd	nd	nd	nd	nd	nd
γ-Curcumene	5.93	0.72	15.1	2.1	3.87	0.80	0.256	0.04	nd	nd	2.79	0.51	0.267	0.03	0.230	0.02	nd	nd
Germacrene D	7.05	0.85	20.7	1.2	5.71	0.65	0.467	0.11	0.053	0.03	2.49	0.76	nd	nd	nd	nd	nd	nd
Eremophilene	2.96	0.54	6.35	0.43	nd	nd	nd	nd	nd	nd	nd	nd	nd	nd	nd	nd	nd	nd
α-Chamigrene	2.65	0.28	6.78	0.41	1.50	0.16	0.243	0.05	0.045	0.03	1.34	0.40	0.035	<0.01	nd	nd	nd	nd
γ-cadinene	3.82	0.48	10.8	0.6	3.83	0.41	0.590	0.12	0.127	0.05	1.43	0.41	0.089	0.01	nd	nd	nd	nd
unknown SQT #3	0.976	0.08	2.77	0.22	0.124	0.02	nd	nd	nd	nd	0.399	0.07	0.014	<0.01	nd	nd	nd	nd
Thujopsane-2β-ol +unknown SQT #4	8.30	1.04	33.1	4.0	1.91	0.30	nd	nd	nd	nd	nd	nd	nd	nd	nd	nd	nd	nd
Widdrol	0.501	0.04	3.47	0.31	0.113	0.03	nd	nd	nd	nd	nd	nd	nd	nd	0.005	<0.01	nd	nd
10-epi-γ-Eudesmol	0.741	0.05	3.14	0.26	0.707	0.04	0.244	0.05	0.055	0.02	0.255	0.15	0.042	<0.01	nd	nd	nd	nd
Cedren-13-ol, 8-	nd	nd	1.35	0.14	0.511	0.06	0.245	0.07	0.089	0.06	nd	nd	nd	nd	nd	nd	nd	nd

Table S3 VOC emission rate per mycelium area (pmol plate^{-1} cm^{-2} h^{-1}) for all sampling dates (days post inoculation) of nutrient rich and nutrient poor cultures of solitary fungi; s.e.: standard error (n = 5); nd: not detectable.

***Alternaria alternata* solitary |A**

	**nutrient rich (malt extract gelrite)**										**nutrient poor (synthetic nutrient poor gelrite)**							
	3		7		14		21		28		14		21		28		35	
Compounds	days	s.e.	days	s.e.	days	s.e.	days	s.e.	days	s.e.	days	s.e.	days	s.e.	days	s.e.	days	s.e.
Isobutanol	0.796	0.23	0.567	0.08	0.024	<0.01	nd	nd	nd	nd	nd	nd	nd	nd	nd	nd	nd	nd
2-Methyl-1-butanol	1.94	0.60	1.01	0.12	0.040	<0.01	nd	nd	nd	nd	nd	nd	nd	nd	nd	nd	nd	nd
β-Elemene	1.08	0.27	0.330	0.02	0.061	0.01	0.028	<0.01	0.004	<0.01	0.034	<0.01	0.003	<0.01	nd	nd	0.001	<0.01
Di-epi-α-cedrene	1.97	0.48	0.684	0.04	0.137	0.04	0.053	<0.01	0.009	0.01	0.056	0.01	0.005	<0.01	nd	nd	0.001	<0.01
α-Cedrene	2.72	0.66	0.781	0.05	0.186	0.03	nd	nd	0.013	<0.01	nd	nd	0.004	<0.01	nd	nd	0.003	<0.01
β-Cedrene	90.3	22.5	21.6	2.6	4.05	0.43	2.13	0.39	0.181	0.09	2.19	0.52	0.165	0.05	0.007	<0.01	0.052	0.01
Thujopsene	28.1	5.0	14.7	0.6	3.07	0.42	1.46	0.41	0.177	0.12	0.885	0.11	0.299	0.04	0.052	<0.01	0.089	0.02
δ-Elemene	nd	nd	nd	nd	nd	nd	nd	nd	nd	nd	nd	nd	nd	nd	nd	nd	nd	nd
(E)-β-Farnesene	nd	nd	0.561	0.02	0.055	<0.01	0.014	0.01	0.001	<0.01	0.048	<0.01	0.004	<0.01	0.001	<0.01	0.001	<0.01
β-acoradiene	4.66	1.11	1.29	0.10	0.320	0.06	0.173	0.03	0.036	0.01	0.192	0.03	0.017	<0.01	nd	nd	nd	nd
α-Himachalene	4.99	1.02	1.57	0.14	0.376	0.06	0.237	0.04	0.020	<0.01	0.295	0.07	0.021	<0.01	nd	nd	0.008	<0.01
β-Chamigrene	3.64	0.79	1.49	0.14	0.205	0.04	0.057	<0.01	nd	nd	nd	nd	nd	nd	nd	nd	nd	nd
unknown SQT #2	0.110	0.03	0.210	0.02	0.007	<0.01	0.018	<0.01	0.001	<0.01	0.022	<0.01	nd	nd	0.001	<0.01	0.001	<0.01
unknown SQT #1	16.82	4.7	5.771	0.32	1.09	0.21	0.422	0.06	0.057	0.02	0.076	0.01	0.046	0.01	nd	nd	nd	nd
allo-Aromadendrene	3.18	0.77	0.917	0.06	0.123	0.03	0.056	0.01	nd	nd	0.064	0.03	nd	nd	nd	nd	nd	nd
γ-Curcumene	3.37	0.90	1.31	0.24	0.193	0.03	0.084	0.02	nd	nd	0.085	0.03	nd	nd	0.004	<0.01	nd	nd
Germacrene D	4.29	1.02	1.36	0.09	0.253	0.05	0.114	0.03	0.009	<0.01	0.120	0.03	0.012	<0.01	0.001	<0.01	0.004	<0.01
Eremophilene	2.24	0.70	0.508	0.05	nd	nd	nd	nd	nd	nd	nd	nd	nd	nd	nd	nd	nd	nd
α-Chamigrene	1.45	0.38	0.515	0.02	0.066	0.01	0.049	<0.01	0.010	<0.01	0.029	<0.01	0.003	<0.01	nd	nd	0.001	<0.01
γ-cadinene	2.33	0.58	0.757	0.06	0.133	0.03	0.122	0.02	0.025	0.01	0.069	0.02	0.007	<0.01	nd	nd	0.002	<0.01
unknown SQT #3	0.811	0.14	0.284	0.02	0.048	<0.01	nd	nd	0.001	<0.01	nd	nd	0.003	<0.01	nd	nd	0.002	<0.01
Thujopsane-2β-ol +unknown SQT #4	7.10	1.06	3.24	0.22	0.618	0.10	0.139	0.02	0.034	<0.01	nd	nd	nd	nd	0.037	<0.01	0.049	<0.01
Widdrol	0.476	0.08	0.333	0.02	0.045	<0.01	0.003	<0.01	0.001	<0.01	nd	nd	nd	nd	0.005	<0.01	0.001	<0.01
10-epi-γ-Eudesmol	0.612	0.10	0.322	0.02	0.081	<0.01	0.053	<0.01	0.031	<0.01	0.053	<0.01	0.011	<0.01	0.003	<0.01	0.005	<0.01
Cedren-13-ol, 8-	nd	nd	0.186	0.02	0.025	<0.01	0.026	<0.01	0.018	<0.01	nd	nd	nd	nd	nd	nd	nd	nd

***Fusarium oxysporum* solitary F|**

	3				14		21		28		14		21		28		35	
Compounds	days	s.e.	7 days	s.e.	days	s.e.	days	s.e.	days	s.e.	days	s.e.	days	s.e.	days	s.e.	days	s.e.
Isobutanol	0.979	0.29	nd	nd	nd	nd	nd	nd	nd	nd	<0.001	<0.01	nd	nd	nd	nd	nd	nd
2-Methyl-1-butanol	1.38	0.35	0.103	0.08	0.002	<0.01	nd	nd	nd	nd	nd	nd	nd	nd	nd	nd	nd	nd
β-Elemene	0.035	0.02	nd	nd	nd	nd	nd	nd	nd	nd	<0.001	<0.01	<0.001	<0.01	nd	nd	nd	nd
β-Cedrene	0.103	0.03	0.022	<0.01	0.005	<0.01	0.001	<0.01	nd	nd	nd	nd	nd	nd	nd	nd	nd	nd
δ-Elemene	2.63	0.97	0.552	0.13	0.049	<0.01	0.018	<0.01	0.010	<0.01	0.040	0.01	nd	nd	0.004	<0.01	nd	nd
β-acoradiene	0.336	0.08	0.070	<0.01	0.007	<0.01	nd	nd	nd	nd	0.021	<0.01	0.001	<0.01	nd	nd	nd	nd
α-Himachalene	1.22	0.40	0.255	0.04	0.026	<0.01	0.009	<0.01	0.005	<0.01	0.020	<0.01	0.005	<0.01	nd	nd	0.001	<0.01
unknown SQT #2	nd	nd	<0.001	<0.01	nd	nd	<0.001	<0.01	nd	nd	nd	nd	nd	nd	nd	nd	nd	nd
γ-Curcumene	nd	nd	nd	nd	0.003	<0.01	0.002	<0.01	nd	nd	0.001	<0.01	nd	nd	0.002	<0.01	nd	nd
Germacrene D	nd	nd	0.004	<0.01	nd	nd	nd	nd	nd	nd	nd	nd	nd	nd	nd	nd	nd	nd

Table S4 VOC emission rate normalized to the projected mycelium area (pmol plate^{-1} cm^{-2} h^{-1}) of *A. alternata* on nutrient rich and nutrient poor co-cultivations; s.e.: standard error (n = 5); nd: not detectable.

split-plate F|A

	**nutrient rich (malt extract gelrite)**										**nutrient poor (synthetic nutrient poor gelrite)**							
Compounds	3 days	s.e.	7 days	s.e.	14 days	s.e.	21 days	s.e.	28 days	s.e.	14 days	s.e.	21 days	s.e.	28 days	s.e.	35 days	s.e.
Isobutanol	0.872	0.10	0.203	0.03	nd	nd	nd	nd	nd	nd	nd	nd	nd	nd	nd	nd	nd	nd
2-Methyl-1-butanol	1.56	0.23	0.325	0.05	nd	nd	nd	nd	nd	nd	nd	nd	nd	nd	nd	nd	nd	nd
β-Elemene	0.743	0.10	0.226	0.02	0.018	<0.01	0.004	<0.01	0.002	<0.01	0.031	<0.01	0.002	<0.01	nd	nd	nd	nd
Di-epi-α-cedrene	1.39	0.17	0.515	0.06	0.030	<0.01	nd	nd	0.009	<0.01	0.048	<0.01	0.002	<0.01	nd	nd	0.002	<0.01
α-Cedrene	1.92	0.23	0.648	0.06	0.053	<0.01	0.011	<0.01	0.003	<0.01	nd	nd	0.003	<0.01	nd	nd	0.001	<0.01
β-Cedrene	63.8	7.7	19.2	1.5	1.31	0.32	0.137	0.07	0.028	0.02	1.80	0.28	0.071	<0.01	nd	nd	nd	nd
Thujopsene	21.1	3.3	10.8	0.6	1.06	0.32	0.168	0.11	0.035	0.02	0.717	0.09	0.088	<0.01	0.004	<0.01	0.001	<0.01
δ-Elemene	0.606	0.09	0.241	0.04	0.009	<0.01	0.022	<0.01	0.012	<0.01	0.131	0.02	nd	nd	0.019	<0.01	nd	nd
(E)-β-Farnesene	nd	nd	0.357	0.03	0.001	<0.01	nd	nd	0.001	<0.01	0.046	0.01	0.001	<0.01	nd	nd	nd	nd
β-acoradiene	3.41	0.41	1.10	0.10	0.100	0.02	0.020	<0.01	0.015	<0.01	0.216	0.05	0.008	<0.01	nd	nd	nd	nd
α-Himachalene	3.32	0.34	1.05	0.11	0.158	<0.01	0.021	<0.01	0.009	<0.01	0.302	0.03	0.011	<0.01	0.011	<0.01	0.005	<0.01
β-Chamigrene	2.41	0.31	0.901	0.05	nd	nd	nd	nd	nd	nd	nd	nd	nd	nd	nd	nd	nd	nd
unknown SQT #2	0.078	<0.01	0.141	0.01	0.002	<0.01	0.001	<0.01	<0.001	<0.01	0.019	<0.01	<0.001	<<0.01	0.001	<0.01	nd	nd
unknown SQT #1	11.6	1.6	3.74	0.54	0.116	0.01	0.053	<0.01	nd	nd	0.251	0.15	0.015	<0.01	nd	nd	nd	nd
allo-Aromadendrene	2.19	0.26	0.658	0.06	0.014	<0.01	nd	nd	nd	nd	0.086	0.04	nd	nd	nd	nd	nd	nd
γ-Curcumene	2.19	0.29	0.723	0.09	0.056	0.02	0.004	<0.01	nd	nd	0.137	0.03	nd	nd	0.006	<0.01	nd	nd
Germacrene D	2.95	0.34	0.970	0.08	0.062	0.02	0.005	<0.01	0.001	<0.01	0.099	0.01	0.004	<0.01	<0.001	<0.01	nd	nd
Eremophilene	1.03	0.18	0.408	0.02	nd	nd	nd	nd	nd	nd	nd	nd	nd	nd	nd	nd	nd	nd
α-Chamigrene	1.03	0.13	0.331	0.03	0.022	<0.01	0.003	<0.01	0.001	<0.01	0.031	0.01	0.001	<0.01	nd	nd	nd	nd
γ-cadinene	1.57	0.19	0.514	0.04	0.054	0.01	0.008	<0.01	0.003	<0.01	0.062	<0.01	0.003	<0.01	nd	nd	<0.001	<0.01
unknown SQT #3	0.54	0.09	0.144	0.01	0.002	<0.01	nd	nd	nd	nd	nd	nd	0.001	<0.01	nd	nd	nd	nd
Thujopsane-2β-ol +unknown SQT #4	4.80	0.88	2.10	0.24	0.050	0.01	nd	nd	nd	nd	nd	nd	nd	nd	nd	nd	nd	nd
Widdrol	0.328	0.05	0.172	0.01	0.001	<0.01	nd	nd	nd	nd	nd	nd	nd	nd	nd	nd	nd	nd
10-epi-γ-Eudesmol	0.423	0.07	0.189	0.02	0.021	<0.01	0.006	<0.01	0.002	<0.01	nd	nd	0.004	<0.01	0.001	<0.01	nd	nd
Cedren-13-ol, 8-	nd	nd	0.160	0.02	0.017	<0.01	0.002	<0.01	0.003	<0.01	nd	nd	nd	nd	nd	nd	nd	nd

direct confrontation FA

Compounds	3 days	s.e.	7 days	s.e.	14 days	s.e.	21 days	s.e.	28 days	s.e.	14 days	s.e.	21 days	s.e.	28 days	s.e.	35 days	s.e.
Isobutanol	1.15	0.16	0.292	0.07	nd	nd	nd	nd	nd	nd	nd	nd	nd	nd	nd	nd	nd	nd
2-Methyl-1-butanol	2.01	0.24	0.507	0.13	nd	nd	nd	nd	nd	nd	nd	nd	nd	nd	nd	nd	nd	nd
β-Elemene	0.870	0.07	0.290	<0.01	0.046	<0.01	0.013	<0.01	0.003	<0.01	0.148	0.04	0.003	<0.01	nd	nd	nd	nd
Di-epi-α-cedrene	1.58	0.14	0.682	0.02	0.116	0.02	0.016	<0.01	0.038	0.02	0.305	0.09	0.003	<0.01	nd	nd	0.002	<0.01
α-Cedrene	2.25	0.21	0.837	0.03	0.155	0.02	0.035	<0.01	0.007	<0.01	nd	nd	0.005	<0.01	nd	nd	0.002	<0.01
β-Cedrene	72.8	6.8	24.7	0.7	4.27	0.55	0.551	0.11	0.072	0.04	13.0	3.8	0.134	0.02	nd	nd	nd	nd
Thujopsene	19.3	3.0	12.0	0.7	2.86	0.25	0.435	0.07	0.055	0.03	1.62	0.61	0.065	<0.01	nd	nd	0.001	<0.01
δ-Elemene	1.88	0.22	0.783	0.05	0.019	<0.01	0.022	<0.01	0.009	<0.01	0.292	0.07	nd	nd	0.022	<0.01	nd	nd
(E)-β-Farnesene	0.550	0.08	0.361	0.01	nd	nd	nd	nd	0.001	<0.01	0.230	0.08	0.002	<0.01	0.001	<0.01	nd	nd
β-acoradiene	3.94	0.36	1.42	0.04	0.257	0.03	0.046	<0.01	0.027	0.01	0.868	0.20	0.012	<0.01	nd	nd	nd	nd
α-Himachalene	6.99	1.86	1.31	0.04	0.491	0.08	0.051	0.01	0.018	<0.01	1.63	0.39	0.018	<0.01	0.026	<0.01	0.009	<0.01
β-Chamigrene	2.30	0.29	0.998	0.07	nd	nd	nd	nd	nd	nd	nd	nd	nd	nd	nd	nd	nd	nd
unknown SQT #2	0.087	<0.01	0.144	0.03	0.006	<0.01	0.003	<0.01	<0.001	<0.01	0.107	0.03	<0.001	<0.01	0.001	<0.01	nd	nd
unknown SQT #1	13.6	1.6	5.40	0.14	0.390	0.14	0.105	0.01	0.027	<0.01	0.231	0.07	0.030	<0.01	nd	nd	nd	nd
allo-Aromadendrene	2.52	0.23	0.854	0.02	0.106	0.02	nd	nd	nd	nd	0.454	0.12	nd	nd	nd	nd	nd	nd
γ-Curcumene	2.85	0.26	0.877	0.10	0.132	0.03	0.009	<0.01	nd	nd	0.687	0.08	0.016	<0.01	0.011	<0.01	nd	nd
Germacrene D	3.38	0.32	1.21	0.04	0.192	0.03	0.016	<0.01	0.002	<0.01	0.607	0.16	nd	nd	nd	nd	nd	nd
Eremophilene	1.38	0.15	0.377	0.04	nd	nd	nd	nd	nd	nd	nd	nd	nd	nd	nd	nd	nd	nd
α-Chamigrene	1.29	0.13	0.396	0.01	0.050	<0.01	0.008	<0.01	0.002	<0.01	0.328	0.09	0.002	<0.01	nd	nd	nd	nd
γ-cadinene	1.83	0.17	0.630	0.02	0.128	0.02	0.020	<0.01	0.004	<0.01	0.349	0.09	0.005	<0.01	nd	nd	nd	nd
unknown SQT #3	0.489	0.07	0.164	0.02	0.004	<0.01	nd	nd	nd	nd	0.099	0.01	<0.001	<0.01	nd	nd	nd	nd
Thujopsane-2β-ol +unknown SQT #4	4.31	0.83	2.00	0.35	0.063	<0.01	nd	nd	nd	nd	nd	nd	nd	nd	nd	nd	nd	nd
Widdrol	0.254	0.04	0.207	0.03	0.004	<0.01	nd	nd	nd	nd	nd	nd	nd	nd	<0.001	<0.01	nd	nd
10-epi-γ-Eudesmol	0.374	0.06	0.188	0.03	0.023	<0.01	0.008	<0.01	0.002	<0.01	0.060	0.04	0.002	<0.01	nd	nd	nd	nd
Cedren-13-ol, 8-	nd	nd	0.081	0.01	0.017	<0.01	0.009	<0.01	0.003	<0.01	nd	nd	nd	nd	nd	nd	nd	nd
